# Synthesis, spectral characterization, electron microscopic study and thermogravimetric analysis of a phosphorus containing dendrimer with diphenylsilanediol as core unit

**DOI:** 10.3762/bjoc.6.85

**Published:** 2010-08-11

**Authors:** E Dadapeer, B Hari Babu, C Suresh Reddy, Naga Raju Charmarthi

**Affiliations:** 1 Department of Chemistry, Sri Venkateswara University, Tirupati, Andhra Pradesh, India -517 502; 2 Department of Chemistry, National Cheng Kung University, Tainan -701, Taiwan

**Keywords:** diphenyl silanediol, divergent method, phosphorus containing dendrimer, scanning electron microscopy, Schiff’s base, thermogravimetric analysis

## Abstract

A phosphorus containing dendrimer with a diphenylsilanediol core was synthesized using a divergent method. Several types of reactions were performed on dendrons of several sizes, either at the level of the core or the surface. The giant Schiff’s base macro molecule possesses 12 imine bonds and 8 hydroxy groups on the terminal phenyl groups. The structures of the intermediate compounds were confirmed by IR, GCMS and ^31^P NMR. The final compound was characterized by ^1^H, ^13^C, ^31^P NMR, MALDI-TOF MS and CHN analysis. Scanning electron microscopic and thermogravimetric analysis/differential scanning calorimetric studies were also performed on the final dendritic molecule.

## Introduction

Dendrimers constitute a new class of macromolecules that can offer a potentially defect-free structure for a diverse range of applications [1–2]. Indeed, these highly branched macromolecules possess precise constitutions and a large number of functional groups which confer upon them unique properties [3]. The word was coined from a combination of dendron (Greek = tree) and polymer. Generally, a linear polymer typically consists of an entangled mixture of individual molecular chains, each having only two terminal groups. But a dendrimer, by contrast, has many branches, each with its own end groups, so that each molecule contains a very high number of terminal functional groups. This gives a molecule resembling a tree. Recently, dendrimers and dendrons have been defined in general terms for a class of hyper branched and poly functional macromolecules [4]. They are also called “cascade-type molecules”. Under ideal conditions, the molecular architecture is very uniform. A dendron is one of the major subdivisions of a dendrimer. Thus, one of the major differences between linear polymers and dendrimers is their branched architecture. They can be constructed by two fundamental approaches namely, the divergent and convergent methods. The divergent method, first reported by Vögtle is based on the successive attachment of branching units to the core molecule [5]. As a result of this growth, each subsequent reaction is characterized by the generation of an exponentially increasing number of functional groups on the periphery, so that a large spherical molecule is formed. This is analogous to the burst of exploding firework. In contrast, the convergent approach involves the synthesis of dendrimeric fragments followed by their subsequent addition to the core. This method was initially described by Hawker and Frechet [6]. The synthesis of dendrimers offers the opportunity to generate monodisperse, structure controlled macromolecular architectures [7–9]. Phosphorus containing dendrimers benefit from the global interest in dendrimers, but they have also their own specificities and properties [10]. Recently, a synthesis has been developed and the properties of phosphorus containing dendrimers has been investigated [11] by several strategies of functionalization of the surface [12] or the branches [13] of dendritic compounds. Similarly, phosphorus-containing dendrons [14] are also becoming of interest. Considerable effort has been devoted to the functionalization of the periphery of dendrimers to explore new properties and applications. Indeed, the properties of these compounds mainly depend on the type of functional end groups they bear. The type of end groups most generally used for further functionalization is limited and are most often –NH_2_, –CHO and –OH groups. One of the common properties of all these end groups is that they are able to react with large range of easily available functionalized compounds. The other property shared by all these end groups is that they are directly obtained during the normal process of synthesis of dendrimers at each generation. To expand the type of functional groups on the periphery of dendrimers, it is desirable to introduce new types of end groups possessing a high potential of reactivity, even if they are not directly available from the normal process. Ideally, the functionalization should be done in a single easy step, without any modification of the general methods used for the synthesis and the growth of the dendrimers. Accordingly, we turned our attention toward the use of –NH_2_, –CHO and –OH end groups on the surface of dendrimers. Amino groups react easily with aldehydes to form Schiff’s bases. Similarly, the phenolic –OH groups react easily and rapidly with P-chlorides and this has the advantage that the only byproduct of the reaction is HCl which can be easily removed during the purification step. Thus, we envisaged that –NH_2_, –CHO and –OH end groups would offer a large number of possibilities to graft new functional groups. The type of dendrimers prepared in the present work is based on the first method of synthesis and involves the condensation reaction between aldehydes and amines. Depending on the step considered, these dendrimers have either aldehyde or –NH_2_, or –OH as reactive end groups. From our previous research on tailored dendrimers [15], we reasoned that the divergent method is a more convenient approach to synthesize phosphorus containing dendrimers [16], since this has the advantage of providing maximum control on the growth of the dendrimer, and macromolecules and a higher number of generations can be obtained.

## Results and Discussion

**Scheme 1:**
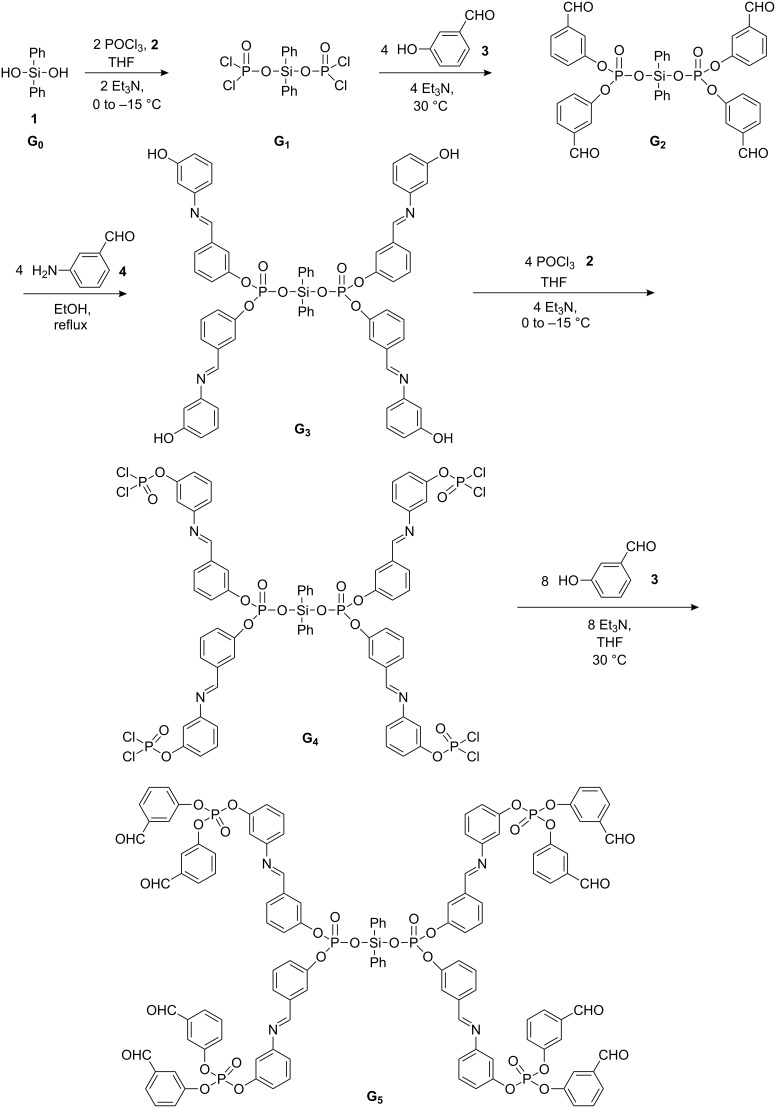
Synthesis of **G_5_**.

**Scheme 2:**
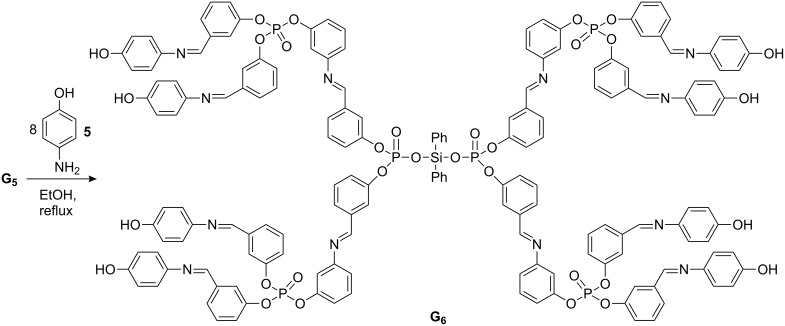
Synthesis of dendritic macromolecule **G_6_**.

Scheme 1 and Scheme 2 summarise the preparation of the dendrimer **G_6_** with diphenyl silanediol as the core unit. The first step of this synthesis is a condensation reaction between diphenyl silanol and POCl_3_ in the presence of triethylamine at 0 to −15 °C to afford the product **G_1_**. In the second step, **G_1_** was treated with 3-hydroxybenzaldehyde at 30 °C in dry THF in presence of triethylamine to form **G_2_** which was then reacted with 3-aminophenol in EtOH at reflux temperature to afford **G_3_**. Compound **G_3_** was treated with POCl_3_ in dry THF in the presence of triethylamine at 0 to −15 °C as before to yield **G_4_**. **G_4_** was then treated with 3-hydroxybenzaldehyde in dry THF at 30 °C to give **G_5_**. Finally, **G_5_** was reacted with 4-aminophenol in dry EtOH at reflux temperature to afford the dendrimer **G_6_**. As the size of the dendron increases the reaction is less effective and the yields are reduced. To improve the yield of **G_6_**, the reaction was carried out in excess ethanol at high temperature. Scheme 1 and Scheme 2 summarize the preparation of the Vögtle-type – dendrimer, the yield of the final compound (**G_6_**) was moderate 65%. The formation of all the intermediates **G_1_**, **G_2_**, **G_3_**, **G_4_** and **G_5_** were characterized by IR, GC-MS and ^31^P NMR data. Their data is given in the Experimental Details, Supporting Information File 1. The synthetic and analytical data of the dendrimer **G_6_** is also given in the Experimental Details, Supporting Information File 1. Compound **G_6_** exhibited absorption bands for Ar–OH, P=O and CH=N in the regions 3360, 1307, and 1616 cm^−1^, respectively. Two absorption bands for P–O–C (aromatic) were found at 970 and 1197 cm^−1^ [17]. In the ^1^H NMR spectra (500 MHz) of **G_6_**, the aromatic protons gave multiplets at δ = 6.01–6.88, the aromatic –OH protons resonated at δ 10.21 as singlet and the imine CH protons appeared as singlets at δ 8.69 and 9.04. The ^13^C NMR spectrum for **G_6_** (see Supporting Information File 2) showed the aromatic carbons at δ = 115.1–156.9 and the methine carbon at δ = 170.8. Compounds **G_4_**, **G_5_** and **G_6_** showed two ^31^P resonance signals indicating two different types of phosphorus atoms.

The mass spectral properties of the final dendrimer **G_6_** was studied by Matrix Assisted Laser Desorption/Ionisation (MALDI) mass spectrometry. As expected, the mass obtained from the MALDI measurements was in good agreement with the calculated value (Table 1).

**Table 1:** Mass data of **G_6_** obtained from MALDI.

Dendrimer	Calculated mass	MALDI mass (MH^+^)

**G_6_**	3038.7	3039.8

The surface topography of the molecule was studied by scanning electron microscopy (SEM), whilst the molecular decomposition of the dendrimer **G_6_** was investigated by both thermogravimetric analysis (TGA) and differential thermal analysis (DTA).

### Scanning electronic microscopic study

**Figure 1:**
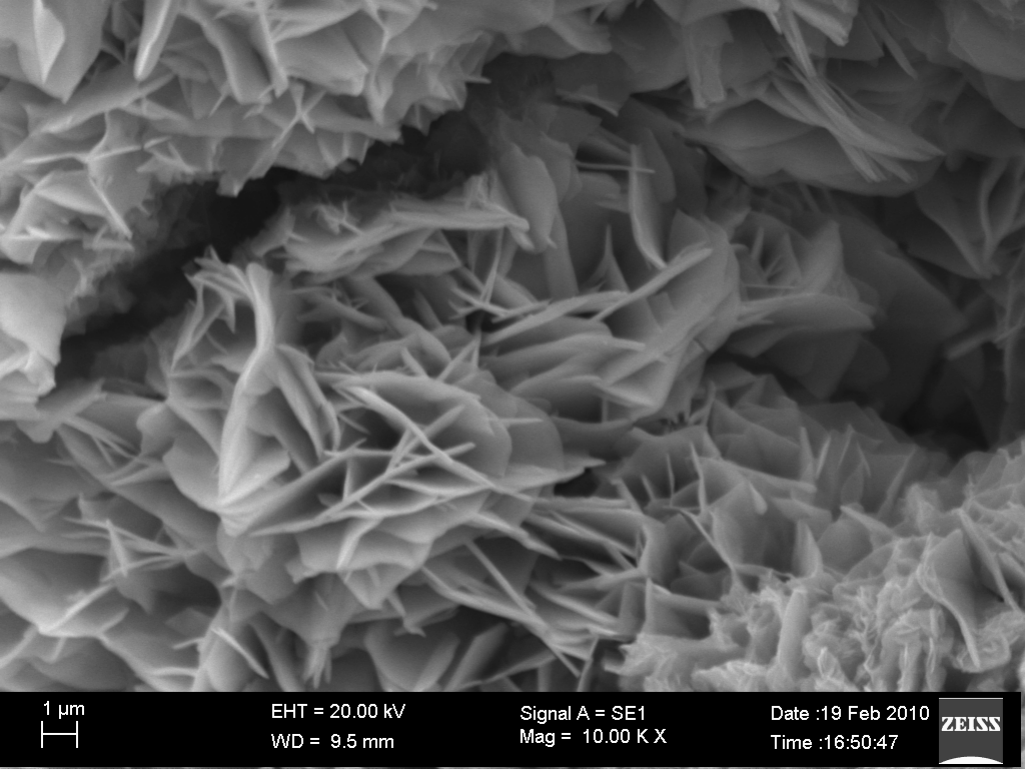
SEM image of **G_6_**.

In order to get a deeper insight into the properties of the surface of our phenyl –OH terminated dendrimer, it was examined by SEM. It revealed that highly resolved patterns with a line width of 1 µm. Figure 1 indicates the dispersion of layered polymeric form of the molecule. The most widely used layered material has negative charges or basic sites on the surface.

### TGA-DTA analysis

**Figure 2:**
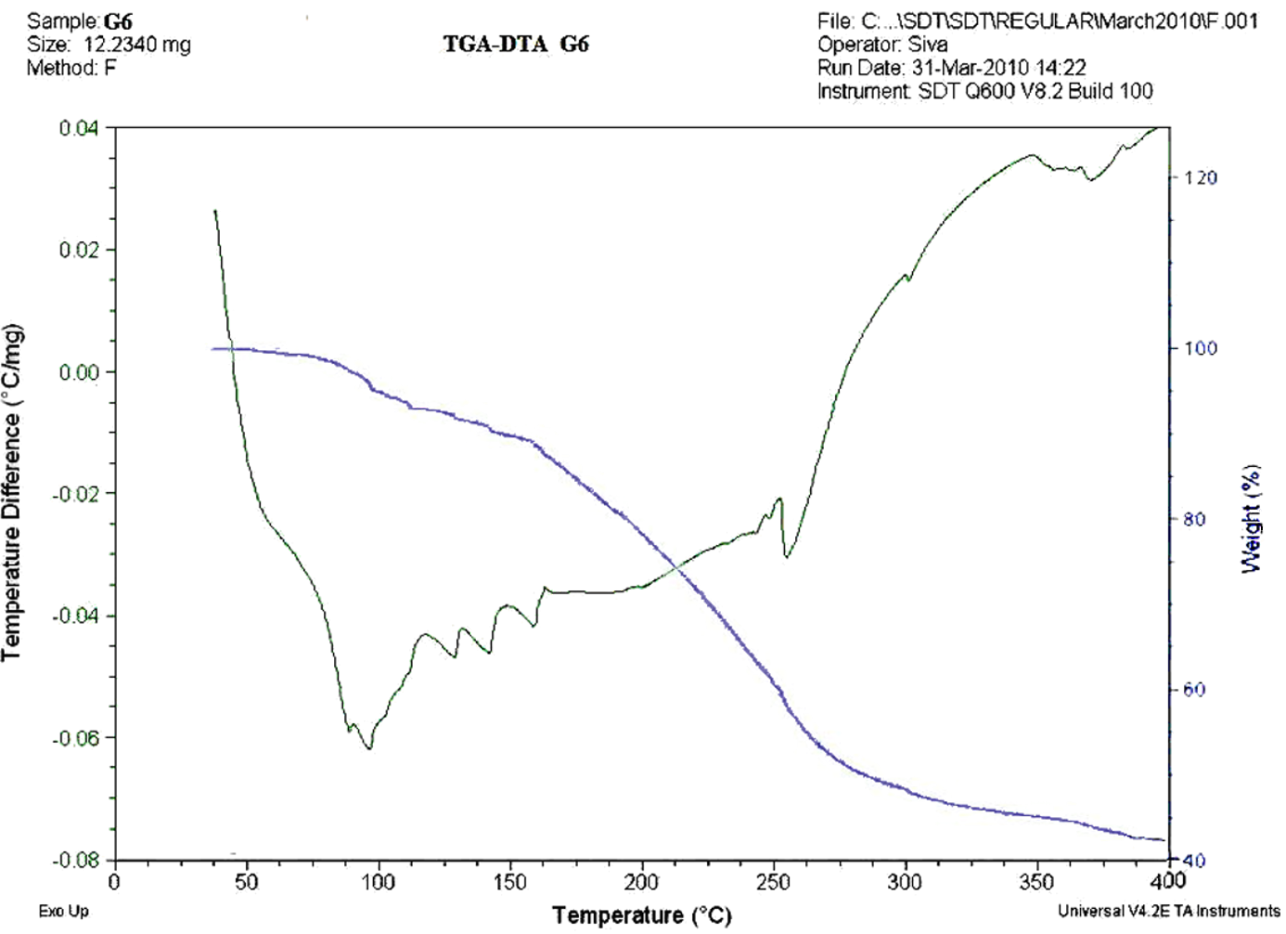
TGA and DTA curve of **G_6_**.

Thermogravimetric analysis (thermal gravimetric analysis (TGA)) was carried out on **G_6_** to determine weight changes with respect to changes in temperature. Similarly, differential thermal analysis (DTA), was performed on **G_6_** to establish whether the changes were either exothermic or endothermic. Simultaneous TGA-DTA measures both heat flow and weight changes (TGA) in a material as a function of temperature in a controlled atmosphere. The TGA-DTA analysis of the compound was recorded up to 400 °C. The TGA graph shows that the compound is stable up to 85 °C and minor decomposition starts at around 90 °C with a corresponding weight loss of approximately 4% (loss of water) and continues up to 165 °C. The calculated water loss is 3.5%. At 165 °C decomposition is more pronounced with a corresponding observed weight loss of approximately 50% up to around 270 °C. This is attributed due to loss of 3-((4-hydroxyphenylimino)methylene)phenoxy groups. The calculated weight loss is 51%. The end-product is stable as indicated by the plateau in the thermogram. An overlay of TGA and DTG plots for compound **G_6_** up to 400 °C is shown in Figure 2. The TGA and DTA studies on **G_6_** were carried out in air. From the DTA curve, the heat of reaction was calculated. The DTA profile (Figure 2) shows an endotherm at 90 °C corresponding to the loss of water and further endotherms at 135, 150 and 165 °C, corresponding to the loss of branches from the compound. The endotherm at 270 °C corresponds to the loss of 3-((4-hydroxyphenylimino)methylene)phenoxy group.

## Conclusion

A simple synthesis of a dendritic macromolecule **G_6_** is presented. The final product **G_6_** as well as intermediates **G_1_**, **G_2_**, **G_3_**, **G_4_** and **G_5_** are stable in the range 50–60 °C. The deeper surface topography of the dendritic molecule **G_6_** was observed by SEM. It reveals the dispersion of layered polymeric form of the molecule due to presence of Si at the core. The thermal stability and changes in weight in relation to change in temperature and the heat of the final dendritic molecule **G_6_** were studied by TGA-DTA analysis. It reveals that the compound **G_6_** is stable up to 85 °C, and then decomposition starts at 90 °C. The loss of water (ca. 4% ) takes place up to 165 °C and between 165 °C to 270 °C there is almost a 50% weight loss. From 270 °C to 400 °C decomposition is very slow. The DTA curve reveals the heat change is endothermic for the loss of water; for the 50% decomposition of the compound the heat change is endothermic at around 225 °C. The small endothermic peaks are due to decomposition of branches.

## Supporting Information

File 1 Experimental details

File 2 Spectral details
