# Supplementary material for: Synthesis, spectral characterization, electron microscopic study and thermogravimetric analysis of a phosphorus containing dendrimer with diphenylsilanediol as core unit
Source: Beilstein J Org Chem. 2010 Aug 11;6:726–31. doi: 10.3762/bjoc.6.85 (PMC2956571; doi:10.3762/bjoc.6.85)
Supplement: File 1 — Experimental details [file Beilstein_J_Org_Chem-06-726-s001.pdf]

Supporting Information

for

Synthesis, spectral characterization, electron microscopic study and thermogravimetric analysis of a phosphorus containing dendrimer with diphenylsilanediol as core unit

E. Dadapeer[1], B. Hari Babu[2], C. Suresh Reddy[1] and Naga Raju Charmathi*[1]

[1]Department of Chemistry, Sri Venkateswara University, Tirupati, Andhra Pradesh, India -517 502 and [2]Department of Chemistry, National Cheng Kung University, Tainan -701, Taiwan

Email: Naga Raju Charmathi - rajuchamarthi10@gmail.com

*Corresponding author

Experimental details

General

All the reagents used in this study were purchased from Sigma-Aldrich Chemical Company and used without further purification. THF and EtOH were dried by standard methods. TLC was performed on pre-coated plates with silica gel $60F_{254}$ (Merk). Column chromatography was performed on silica gel (0.040–0.063 mm, Macherey Nagel).The melting points were determined on a Buchi R-535 melting point apparatus and are uncorrected. IR Spectra were recorded on JASCO FT IR-5300 Spectrometer at University of Hyderabad using KBr optics. ^{1}H and ^{13}C NMR spectra were recorded on a Bruker A VIII 500 MHz NMR spectrometer at IIT, Chennai, operating at 500.13 MHz for ^{1}H, 125.75 MHz for ^{13}C and 202 MHz for ^{31}P NMR. NMR data were recorded in DMSO-d_6 and chemical shifts were referenced to TMS (^{1}H and ^{13}C) and 85% H_3PO_4 (^{31}P). Mass spectra of intermediate dendrons were recorded on JEOL GCMate at IIT, Chennai. MALDI spectrum of final dendrimer was recorded using an Applied Biosystems MALDI-TOF Voyager De-Pro spectrometer. The sample was run using Sinapic acid as the matrix with DMSO as the solvent in the dried-droplet preparation method at IIT Chennai. TGA-DTA measurement was taken using a SDT Q600 V8.2 Build instrument, performed at IISc, Bangalore. Scanning Electron Microscopy (SEM) images were taken with a Carl Zeiss, EVO MA15 Instrument. SEM operated at 20 kVat the Department of Physics, S. V. University, Tirupati, India. Elemental analyses were performed using EA 1112 Thermo Finnigan instrument at University of Hyderabad, Hyderabad, India.

General procedure for Synthesis of dendrimer, G6

Preparation of G_1

A solution of $POCl_3$ (**2**) (0.01 mol, 0.93 mL) in 20 mL of dry THF was added dropwise over a period of 20 min to a stirred solution of diphenylsilanediol (**1**) (0.005 mol, 1.08 g) in 25 mL of dry THF and triethylamine (0.01 mol) at 0 to −15 °C. After stirring for 3 h at 30–40 °C,

formation of $\mathbf{G_1}$ was confirmed by TLC (average R_f = 0.75, 3:7 mixture of ethyl acetate and hexane). Triethylamine hydrochloride was removed by filtration and the product characterized by IR, GC-MS and ^{31}P spectral data. The solution (filtrate A) was then used for the next reaction step without further purification.

Spectral Data

$\mathbf{G_1}$: Yield 80%, δ_P (202 MHz, 85%, H_3PO_4), −0.85 ppm; *m/z* (GCMS) 440.05 $(M)^+$.

Preparation of G_2

To a stirred solution of the filtrate A and triethylamine (0.02 mol), a solution of 3-hydroxybenzaldehyde (**3**) (0.02 mol, 2.44 g) in 25 mL of dry THF was added dropwise over a period of 20 min at 0 to −15 °C. After stirring for 4 h at 40-45 °C, formation of $\mathbf{G_2}$ was confirmed by TLC (average R_f = 0.65, 4:6 mixture of ethyl acetate and hexane). Triethylamine hydrochloride was removed by filtration. The solvent was removed in a rotary evaporator to yield the crude product. It was purified by column chromatography on silica gel (60–120 mesh), ethyl acetate:hexane (2:8) to afford the pure compound. The compound thus obtained was characterized by IR, GC-MS and ^{31}P spectral data and used for the next reaction step.

Spectral Data

$\mathbf{G_2}$: Yield 73%, υ_{max} (KBr), 1244 (P=O), 981, 1183 (P–O–$C_{aromatic}$), 1697 (Ar–CHO); δ_P (202 MHz, 85%, H_3PO_4), −11.91 ppm; *m/z* (GCMS) 792.19 $(M)^+$.

Preparation of G_3

To a stirred solution of $\mathbf{G_2}$ in 25 mL dry EtOH, a solution of 3-aminophenol (**4**) (0.02 mol, 2.18 g) in 25 mL of dry EtOH, was added at room temperature. After stirring for 5 h at reflux temperature, formation of $\mathbf{G_3}$ was confirmed by TLC (average R_f = 0.63, 6:4 mixture of ethyl acetate and hexane). The solvent was evaporated under reduced pressure to give the crude product which was purified by washing with ethyl acetate and hexane. The imine ($\mathbf{G_3}$) thus

obtained was characterized by IR, GC-MS and ^{31}P NMR spectral data and used for the next step reaction.

Spectral Data

G_3: Yield 70%, υ_{max} (KBr), 1257 (P=O), 962, 1176 (P–O–$C_{aromatic}$), 3362 (Ar-OH), 1602 (CH=N); δ_P (202 MHz, 85%, H_3PO_4), -11.41 ppm; *m/z* (GCMS) 1157.13$(M)^+$.

Preparation of G_4

To a stirred solution of **G_3** and triethylamine (0.02 mol), a solution of $POCl_3$ (**2**) (0.02 mol, 1.87 mL) in 20 mL of dry THF was added dropwise over a period of 20 min at 0–5 °C. After stirring for 4 h at 40–45 °C, formation of G_4 was confirmed by TLC (average R_f = 0.59, 5:5 mixture of ethyl acetate and hexane). Triethylamine hydrochloride was removed by filtration. The solvent was evaporated under reduced pressure to give the crude product which was purified by column chromatography on silica gel (60–120 mesh, ethyl acetate:hexane, 2:8) to afford the pure compound. The compound thus obtained was characterized by IR, GC-mass and ^{31}P NMR spectroscopy and used for the next step reaction.

Spectral Data

G_4: Yield 69%, υ_{max} (KBr), 1261 (P=O), 960, 1186 (P–O–$C_{aromatic}$), 1615 (CH=N); δ_P (202 MHz, 85%, H_3PO_4), −11.49, −5.86 ppm; *m/z* (GCMS) 1624.62 $(M)^+$.

Preparation of G_5

To a stirred solution of the filtrate **G_4** and triethylamine (0.04 mol), a solution of 3-hydroxybenzaldehyde (**3**) (0.04 mol, 4.88 g) in 30 mL of dry THF was added dropwise over a period of 20 min at 0–5 °C. After stirring for 4 h at 40–45 °C, formation of **G_5** was confirmed by TLC (average R_f = 0.55, 7:3 mixture of ethyl acetate and hexane). Triethylamine hydrochloride was removed by filtration and the solvent evaporated under reduced pressure to obtain the crude product. Purification by column chromatography on silica gel (60–120 mesh, ethyl

acetate:hexane, 2:8) gave the pure compound. The compound thus obtained was characterized by IR, GC-MS and ^{31}P NMR spectroscopy and used for the next step reaction.

Spectral Data

G_5: Yield 61%, υ_{max} (KBr), 1277 (P=O), 923, 1202 (P–O–$C_{aromatic}$), 1620 (CH=N), 1660 Ar–CHO; δ_P (202 MHz, 85%, H_3PO_4), −11.92, −18.00 ppm; *m/z* (GCMS) 2309.90 $(M)^+$.

Preparation of G_6

To a stirred solution of **G_5** in 30 mL dry EtOH, a solution of 4-aminophenol (**5**) (0.04 mol, 4.36 g) in 25 mL of dry EtOH, was added at room temperature. After stirring for 5 h at reflux temperature, formation of **G_6** was confirmed by TLC (average R_f = 0.51, 8:2 mixture of ethyl acetate and hexane). The solvent was evaporated under reduced pressure to give the crude product which was purified by washing with ethyl acetate and hexane. The imine thus obtained was characterized by IR, ^{1}H, ^{13}C, ^{31}P NMR, MALDI-TOF-MS and C, H, N analysis.

Spectral Data

G_6: Yield 65%, υ_{max} (KBr), 3360 (Ar–OH), 1307 (P=O), 970, 1197 (P–O–$C_{aromatic}$), 1616 (CH=N), δ_H (500 MHz, DMSO-*d_6*), 10.21 (8H, s, Ar–OH), 6.01–6.88 (64 H, m, Ar–H), 8.69, 9.04 (12H, s, –CH=N); δ_C (125 MHz, DMSO-*d_6*), 129.6 (C_0-1/ C^1_0-1), 128.6 (C_0-2 & C_0-6/ C^1_0-2 & C^1_0-6), 127.3 (C_0-3 & C_0-5/ C^1_0-3 & C^1_0-5), 129.1 (C_0-4/ C^1_0-4), Si-Ar carbons), 147.6 (C_1-1/ C^1_1-1/ C_3-1/ C^1_3-1), 120.8 (C_1-2/ C^1_1-2/ C_3-2/ C^1_3-2), 128.6 (C_1-3/C^1_1-3/ C_4-3/C^1_4-3), 119.8 (C_1-4/ C^1_1-4/ C_3-4/ C^1_3-4), 144.5 (C_1-5/ C^1_1-5/ C_3-5/ C^1_3-5), 115.1 (C_1-6/ C^1_1-6/ C_3-6/ C^1_3-6), P–O–Ar carbons), 154.4 (C_2-1/ C^1_2-1), 111.0 (C_2-2/ C^1_2-2), 147.6 (C_2-3/C^1_2-3), 118.4 (C_2-4/ C^1_2-4), 127.7 (C_2-5/ C^1_2-5), 115.2 (C_2-6/ C^1_2-6), P–O–Ar carbons), 144.9 (C_4-1/ C^1_4-1), 127.3 (C_4-2& C_4-6/ C^1_4-2& C^1_4-6), 119.7 (C_4-3& C_4-5/ C^1_4-3& C^1_4-5), 156.9 (C_0-4/ C^1_0-4), 170.8 (CH=N); δ_P (202 MHz, 85%, H_3PO_4), −12.09, −18.29 ppm; *m/z* (Maldi-TOF) 3039.8 $(MH)^+$; Anal. Calc. for $C_{14}H_{19}N_2O_8P$: C, 66.40; H, 4.18; N, 5.53%; found: C, 66.25; H, 4.22; N, 5.61%.
