# Supplementary material for: Synthesis, spectral characterization, electron microscopic study and thermogravimetric analysis of a phosphorus containing dendrimer with diphenylsilanediol as core unit
Source: Beilstein J Org Chem. 2010 Aug 11;6:726–31. doi: 10.3762/bjoc.6.85 (PMC2956571; doi:10.3762/bjoc.6.85)
Supplement: File 2 — Spectral details [file Beilstein_J_Org_Chem-06-726-s002.pdf]

Supporting Information

for

Synthesis, spectral characterization, electron microscopic study and thermogravimetric analysis of a phosphorus containing dendrimer with diphenylsilanediol as core unit

E. Dadapeer[1], B. Hari Babu[2], C. Suresh Reddy[1] and Naga Raju Charmathi*[1]

[1]Department of Chemistry, Sri Venkateswara University, Tirupati, Andhra Pradesh, India -517 502 and [2]Department of Chemistry, National Cheng Kung University, Tainan -701, Taiwan

Email: Naga Raju Charmathi - rajuchamarthi10@gmail.com

*Corresponding author

Spectral details

G$_1$ Spectra

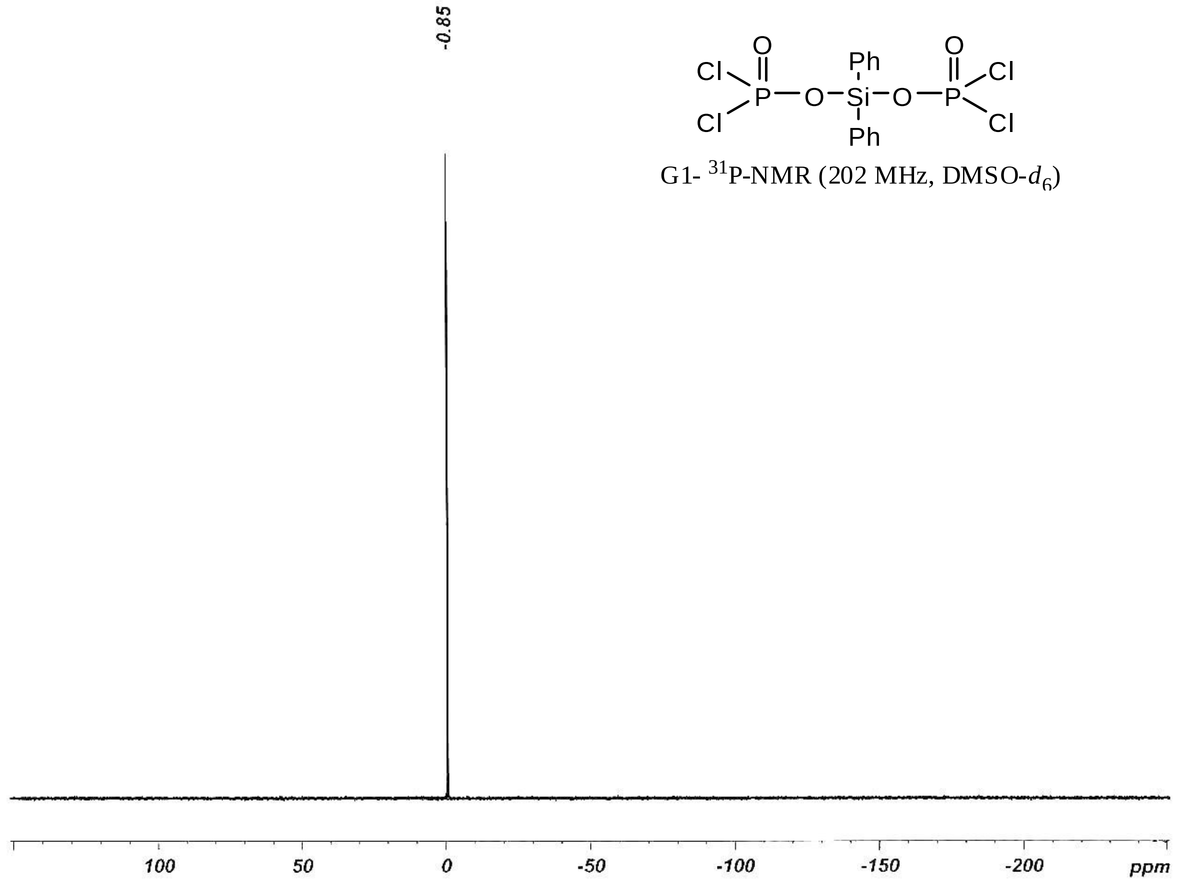

G$_1$ ^{31}P-NMR Spectrum

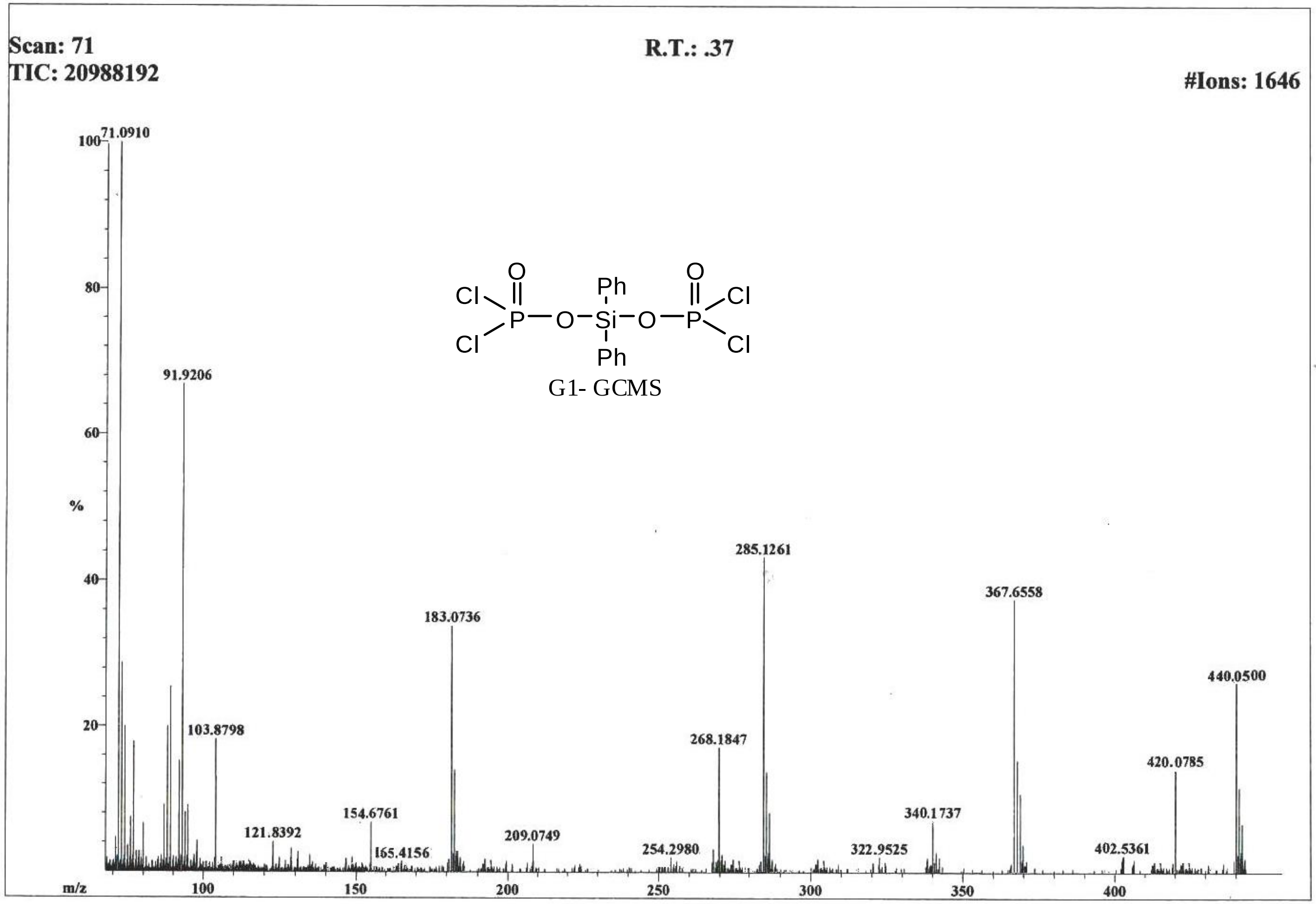

Scan: 71
TIC: 20988192
R.T.: .37
#Ions: 1646
71.0910
91.9206
103.8798
121.8392
154.6761
165.4156
183.0736
209.0749
254.2980
268.1847
285.1261
322.9525
340.1737
367.6558
402.5361
420.0785
440.0500
%
m/z
G1- GCMS

G$_1$ GCMS

G_2 Spectra

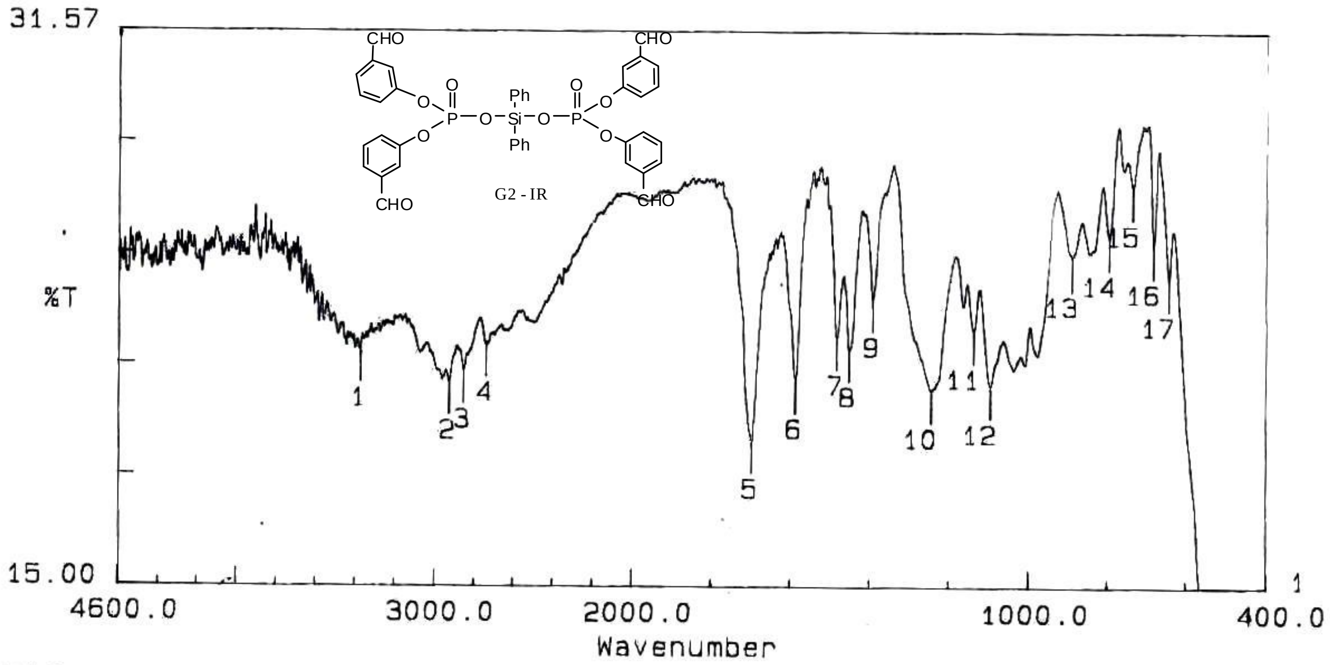

Condition

upper 31.56 lower 15.00 depth 1.00

Peak table

1:	3373.80 (22.1)	2:	2924.35 (21.1)	3:	2851.05 (21.4)	4:	2737.24 (22.2)
5:	1697.51 (19.4)	6:	1587.56 (21.2)	7:	1483.39 (22.5)	8:	1452.53 (22.1)
9:	1392.73 (23.6)	10:	1244.20 (20.9)	11:	1183.10 (22.7)	12:	1095.67 (21.0)
13:	981.19 (24.9)	14:	796.67 (25.5)	15:	736.87 (27.0)	16:	682.86 (25.2)
17:	644.28 (24.3)						

G_2 IR Spectrum

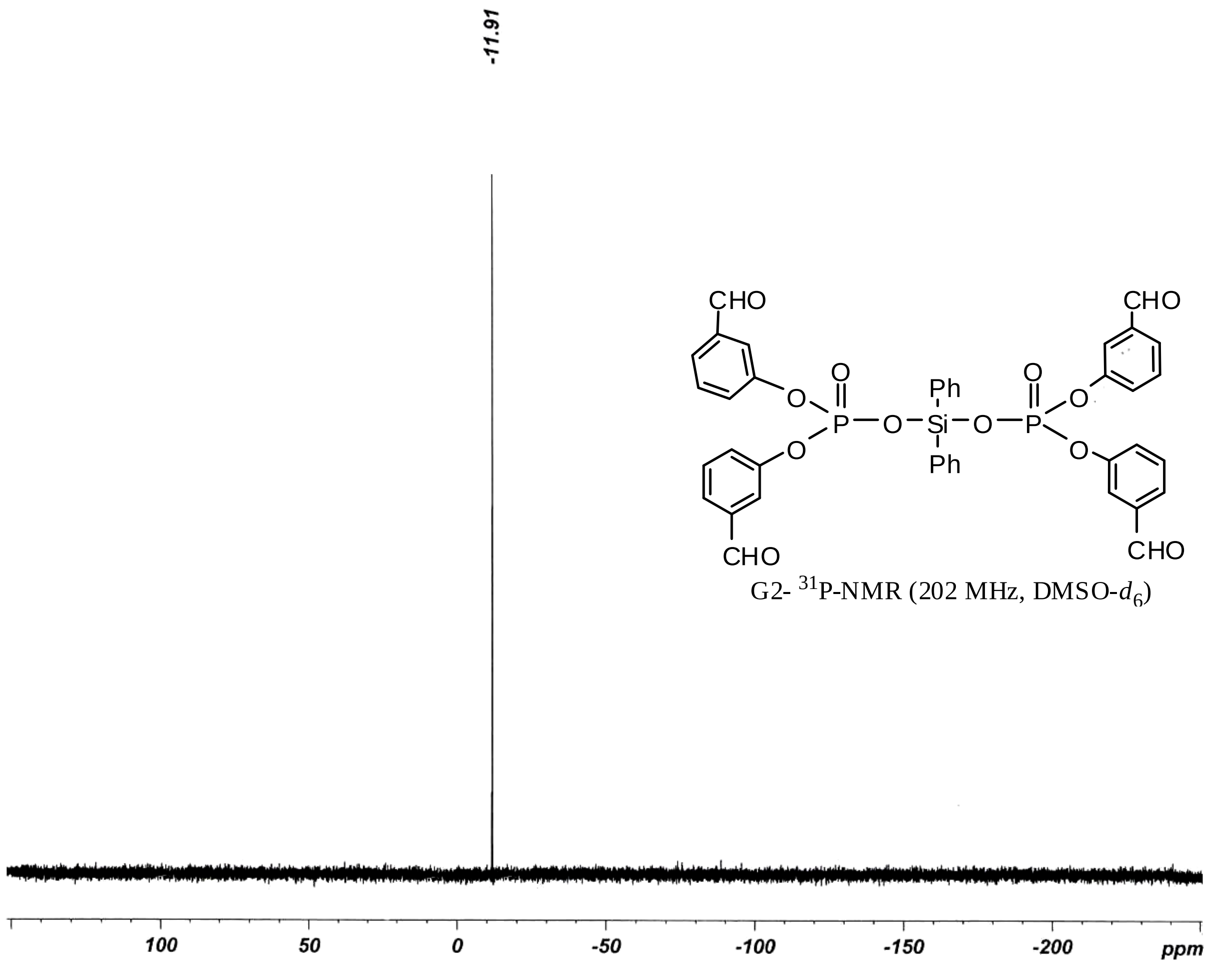

G_2 ^{31}P-NMR Spectrum

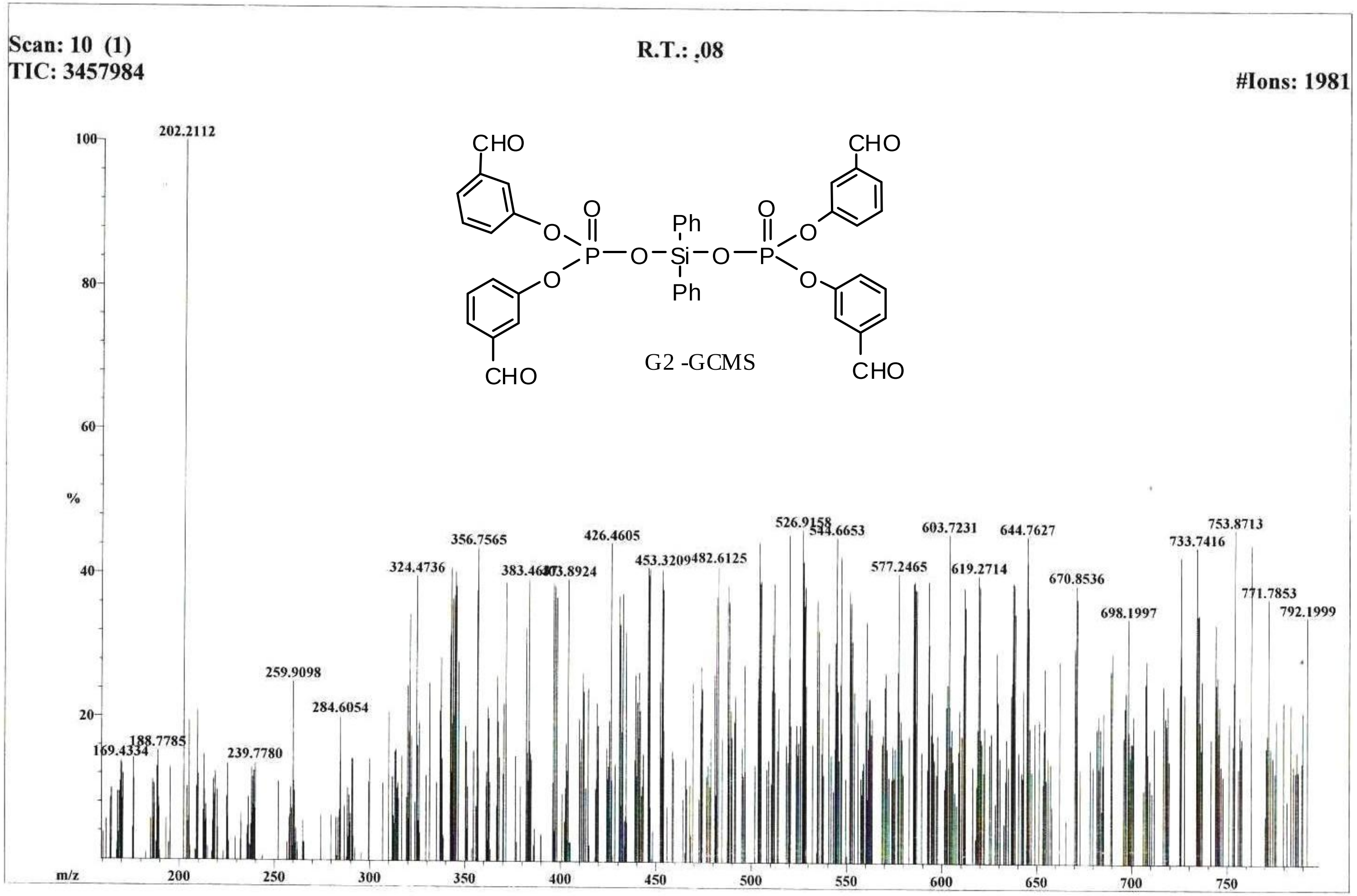
Scan: 10 (1)
TIC: 3457984
R.T.: ,08
#Ions: 1981
CHO
CHO
O
O
Ph
O
P
O
Si
O
P
O
O
Ph
O
CHO
CHO
G2 -GCMS
%
m/z
202.2112
169.4334
188.7785
239.7780
259.9098
284.6054
324.4736
356.7565
383.4637
403.8924
426.4605
453.3209
482.6125
526.9158
544.6653
577.2465
603.7231
619.2714
644.7627
670.8536
698.1997
733.7416
753.8713
771.7853
792.1999

G₂ GCMS

G_3 Spectra

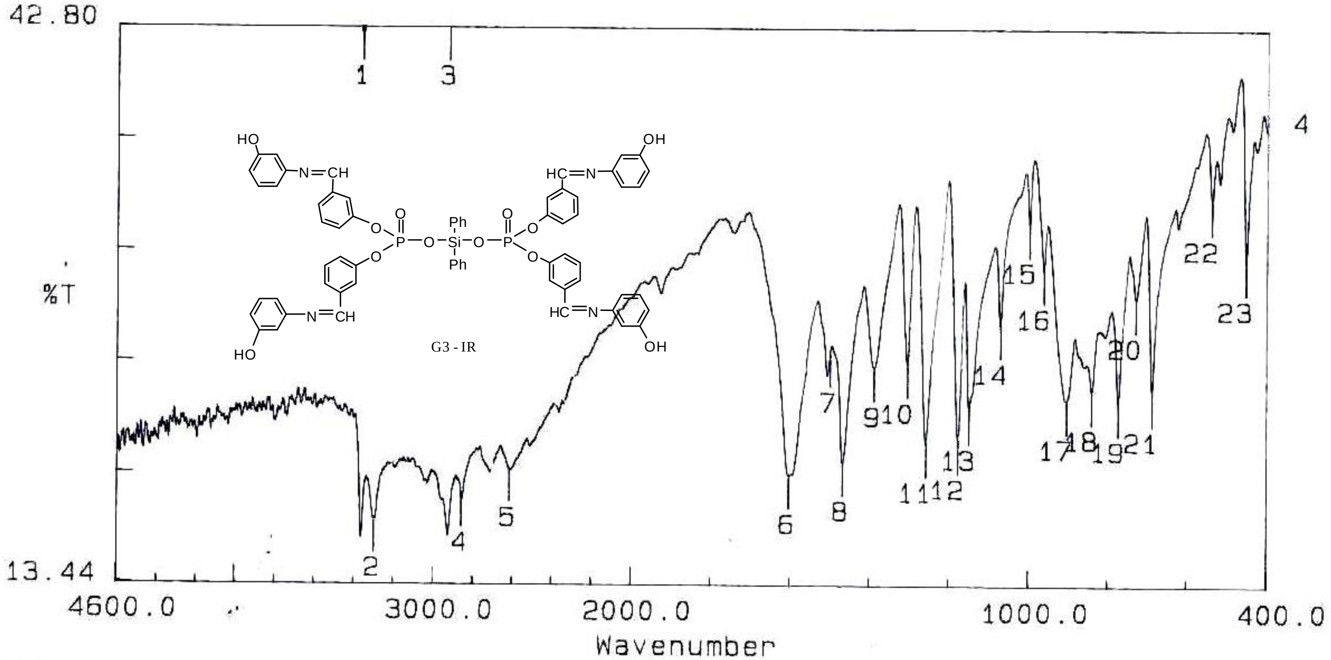

Condition

upper 42.80 lower 13.43 depth 2.00

Peak table

1:	3362.23 (15.9)	2:	3296.64 (16.9)	3:	2924.35 (16.0)	4:	2858.76 (18.3)
5:	2613.78 (19.6)	6:	1602.99 (19.2)	7:	1498.82 (25.7)	8:	1467.96 (20.0)
9:	1388.87 (25.0)	10:	1304.00 (25.1)	11:	1257.70 (21.0)	12:	1176.68 (21.1)
13:	1149.68 (22.7)	14.	1070.59 (27.2)	15:	999.22 (32.5)	16:	962.57 (30.1)
17:	904.70 (23.2)	18:	841.04 (23.7)	19:	773.52 (23.1)	20:	729.16 (28.6)
21:	686.72 (23.6)	22:	538.19 (33.8)	23:	453.31 (30.6)		

G_3 IR Spectrum

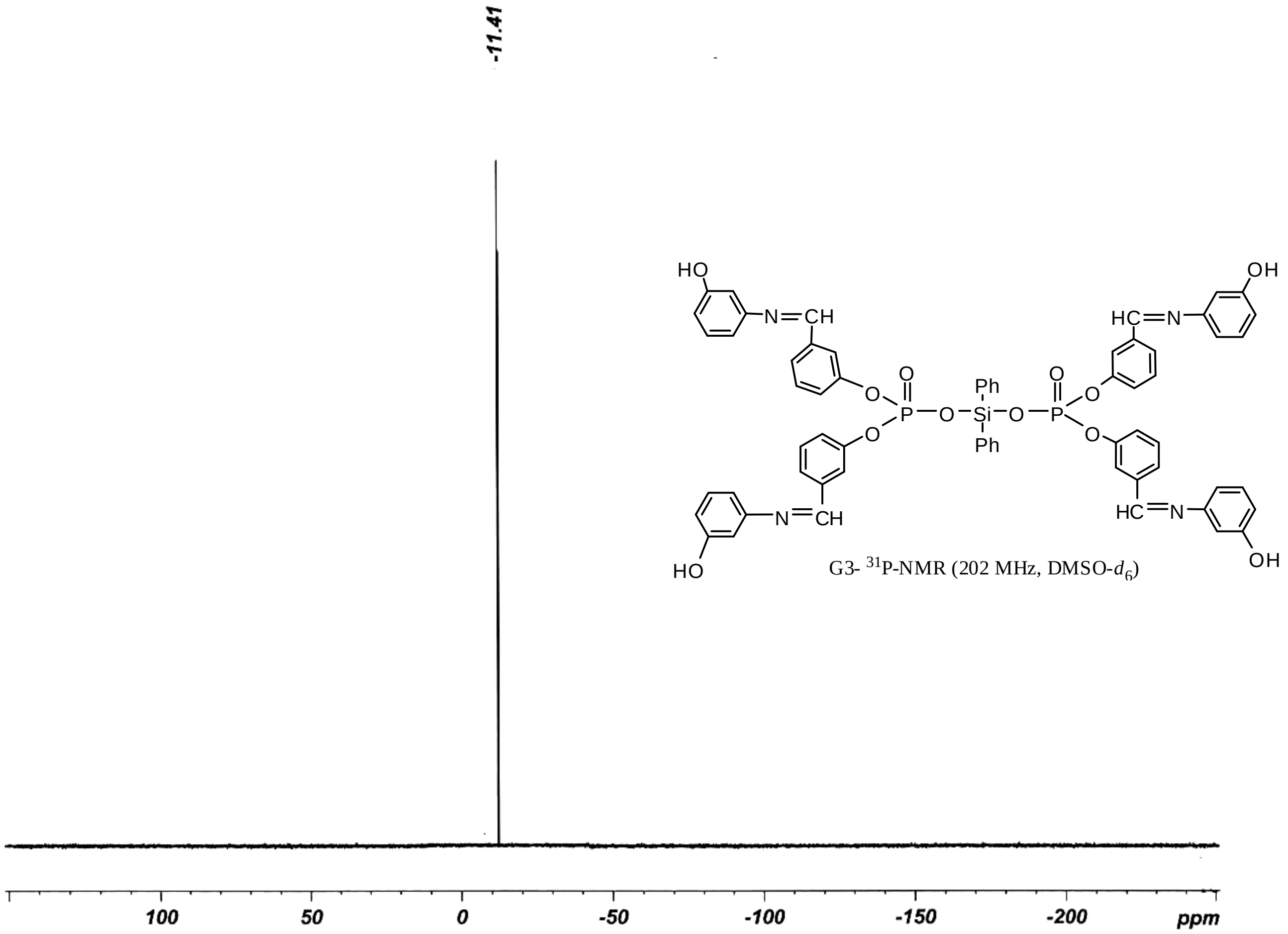

G_3 ^{31}P-NMR Spectrum

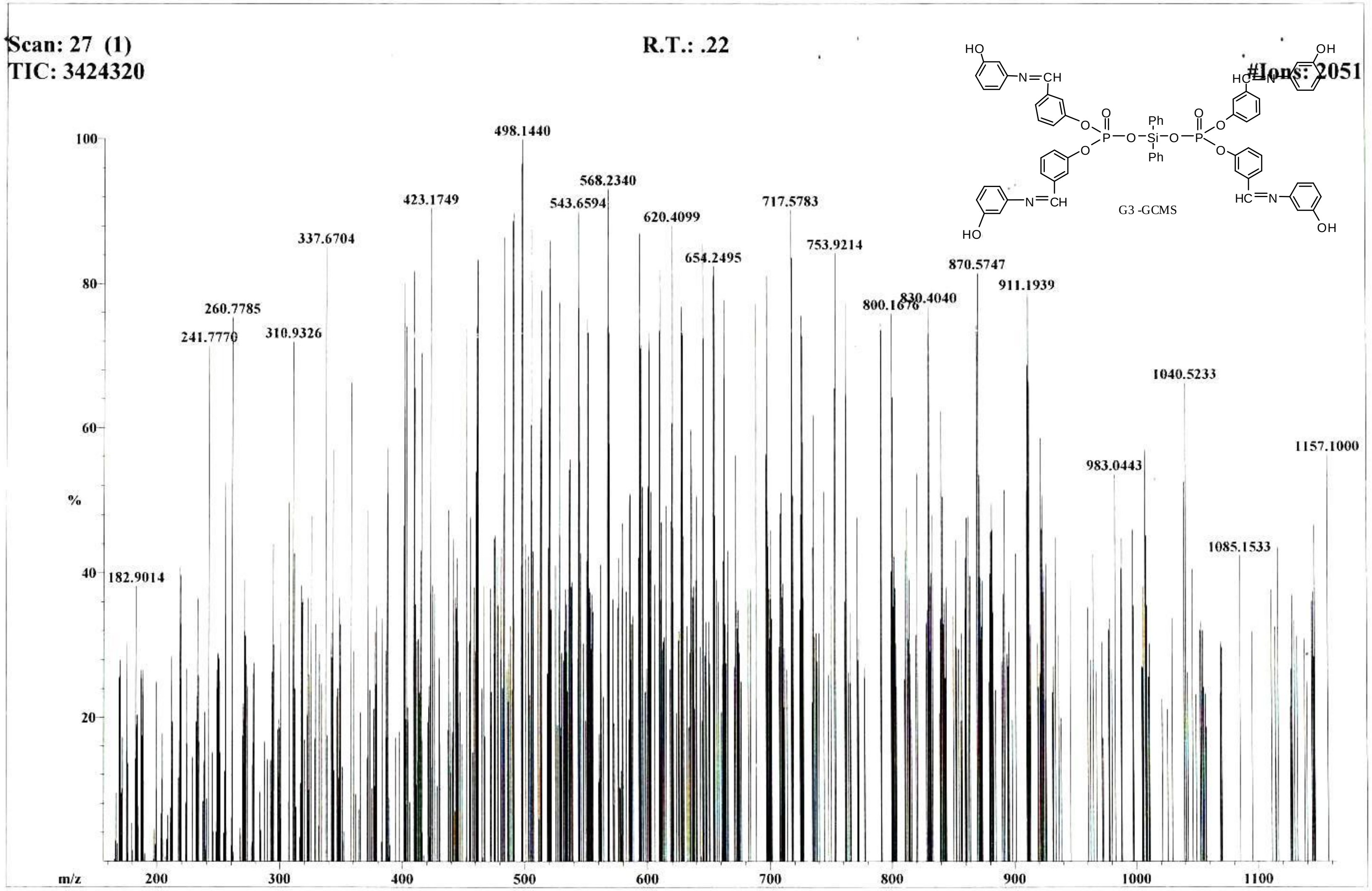
Scan: 27 (1)
TIC: 3424320
R.T.: .22
#Ions: 2051
498.1440
568.2340
543.6594
423.1749
620.4099
717.5783
337.6704
654.2495
753.9214
870.5747
911.1939
260.7785
241.7770
310.9326
800.1676
830.4040
1040.5233
1157.1000
983.0443
1085.1533
182.9014
G3 -GCMS
%
m/z

G$_3$ GCMS

G_4 Spectra

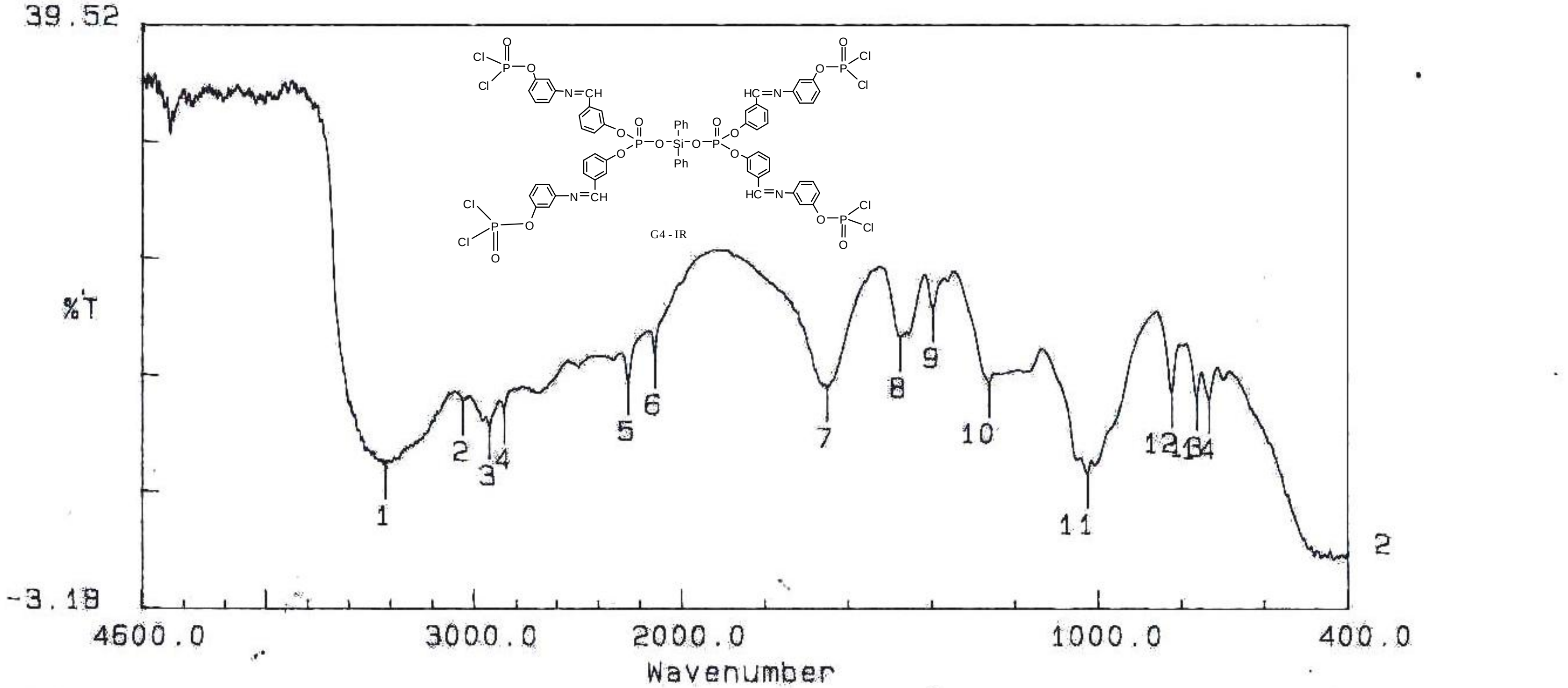

Condition

upper 39.51 lower -3.18 depth 1.00

Peak table

1: 3427.81 (7.4)	2: 3053.59 (12.0)	3: 2926.28 (10.3)	4: 2854.90 (11.5)
5: 2254.99 (13.5)	6: 2127.68 (15.5)	7: 1615.22 (13.0)	8: 1475.68 (16.7)
9: 1396.59 (19.0)	10: 1261.56 (13.4)	11: 1186.22 (6.7)	12: 960.68 (12.7)
13: 861.95 (12.4)	14: 733.02 (12.2)		

G_4 IR Spectrum

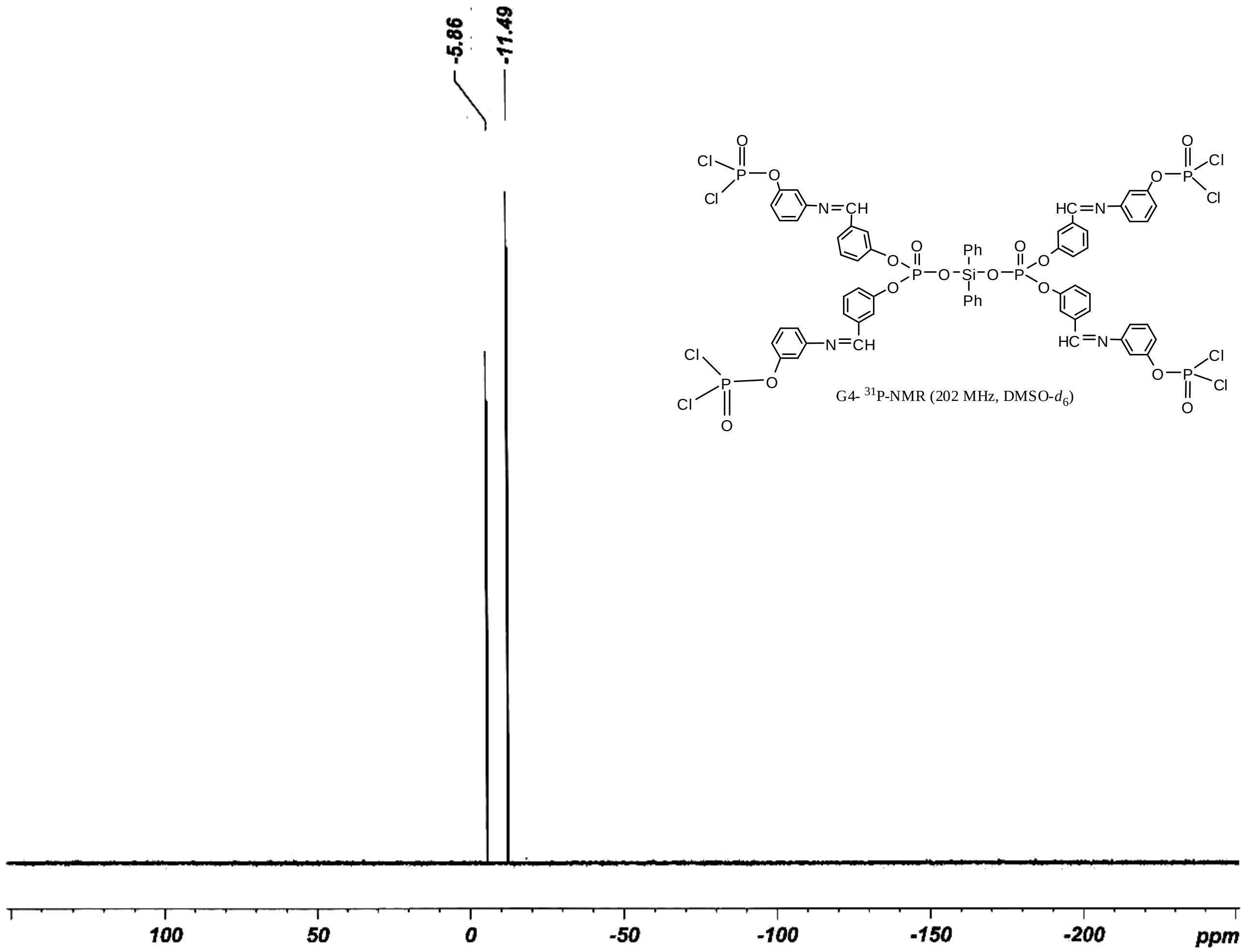

G_4 ^{31}P-NMR Spectrum

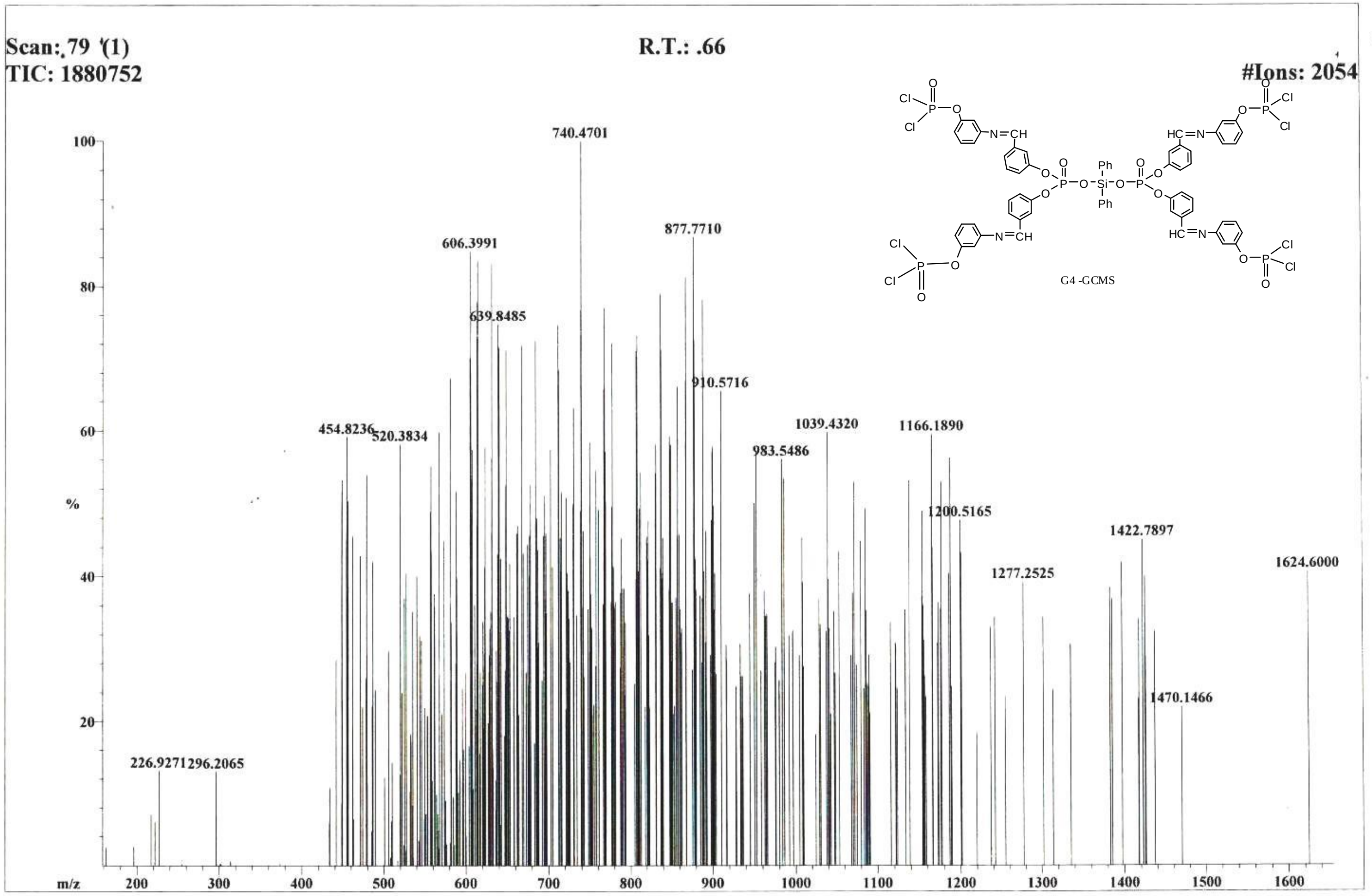
Scan: 79 (1)
R.T.: .66
TIC: 1880752
#Ions: 2054
740.4701
877.7710
606.3991
639.8485
910.5716
454.8236
520.3834
1039.4320
1166.1890
983.5486
1200.5165
1422.7897
1624.6000
1277.2525
1470.1466
226.9271
296.2065
%
m/z
G4 -GCMS

G_4 GCMS

G_5 Spectra

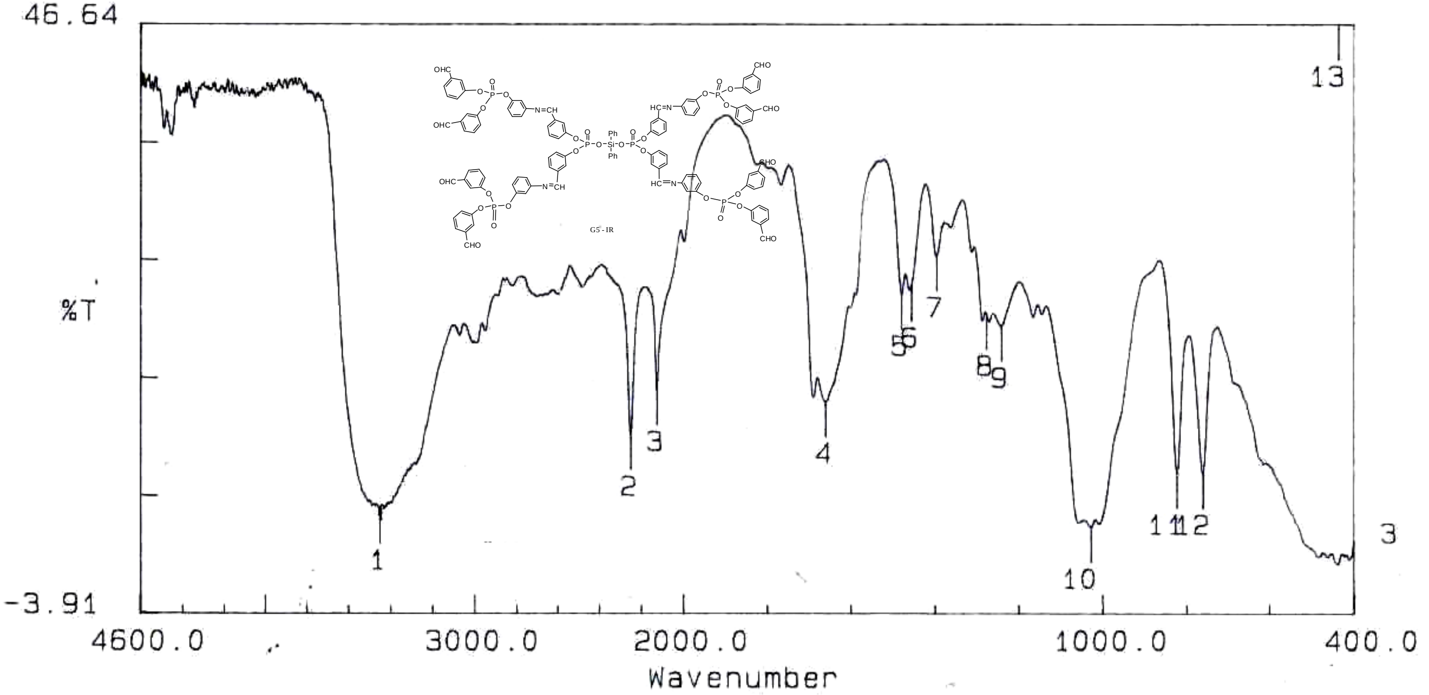

Condition

upper 46.63 lower -3.91 depth 2.00

Peak table

1:	3452.89 (5.1)	2:	2251.13 (11.5)	3:	2125.75 (15.3)	4:	1660.86 (14.3)
5:	1620.53 (23.5)	6:	1456.39 (24.2)	7:	1396.59 (26.8)	8:	1277.00 (21.8)
9:	1202.27 (20.8)	10:	1028.15 (3.5)	11:	923.68 (8.2)	12:	860.02 (8.1)
13:	537.88 (0.3)						

G_5 IR Spectrum

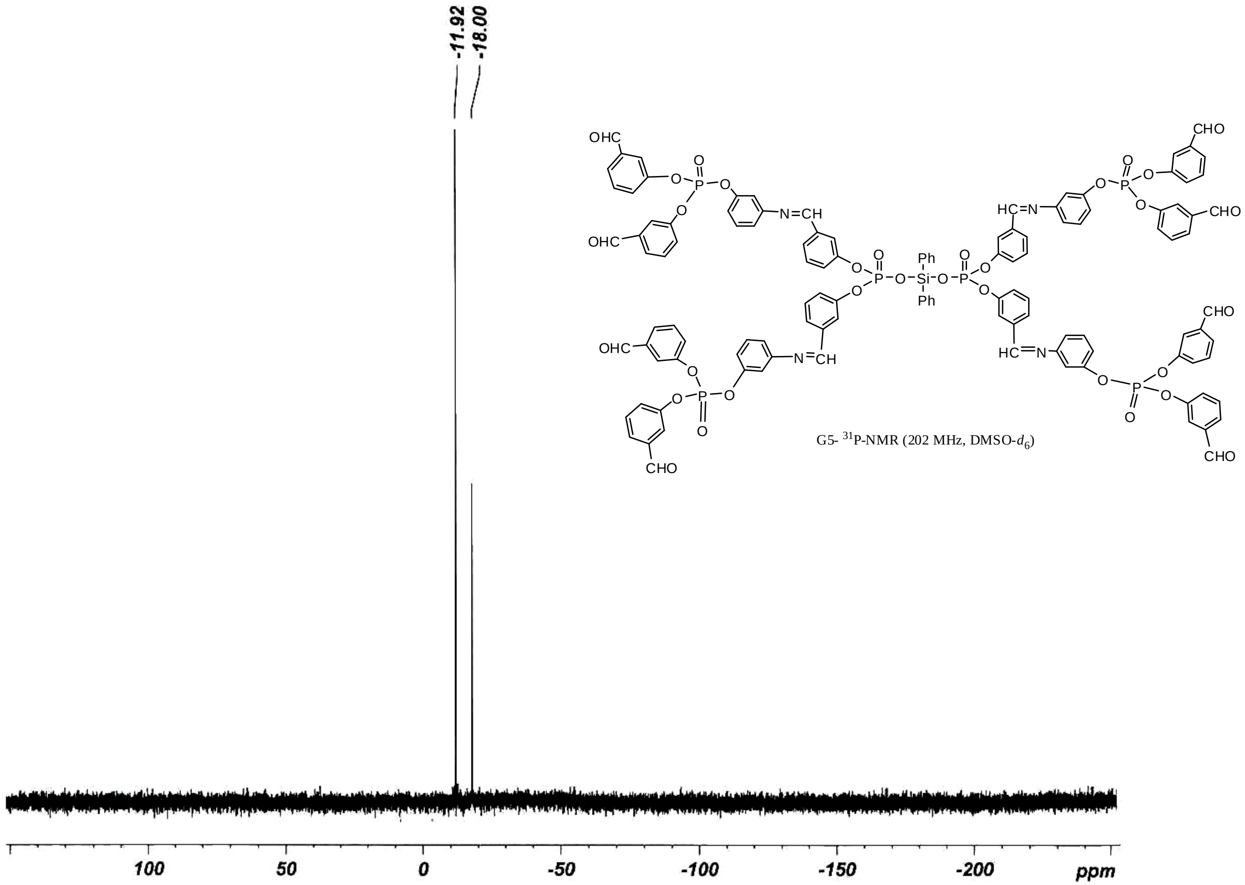

G_5 ^{31}P-NMR Spectrum

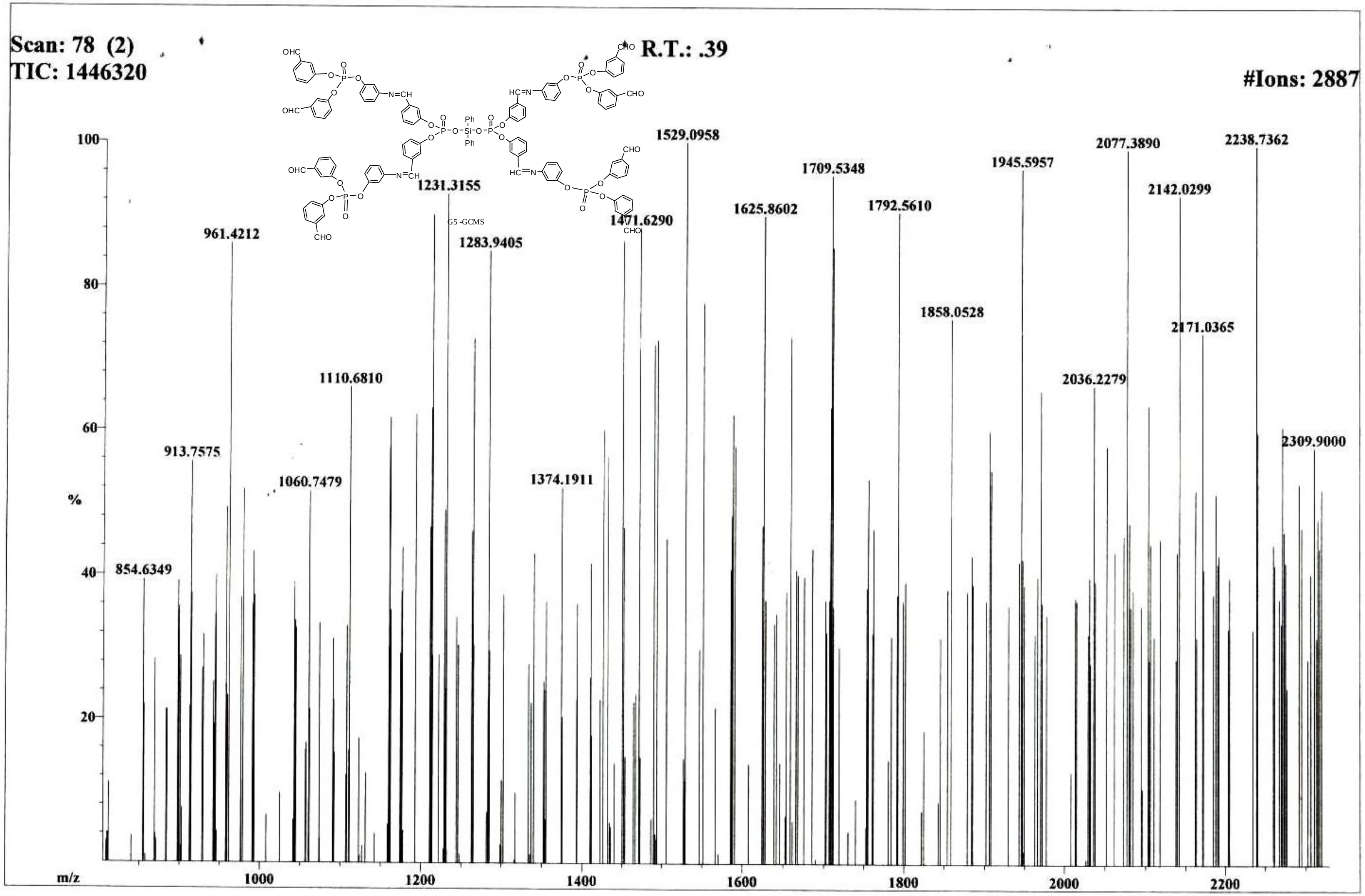
Scan: 78 (2)
TIC: 1446320
R.T.: .39
#Ions: 2887
G5 -GCMS
1529.0958
2077.3890
2238.7362
1709.5348
1945.5957
1231.3155
2142.0299
1625.8602
1792.5610
1471.6290
961.4212
1283.9405
1858.0528
2171.0365
1110.6810
2036.2279
2309.9000
913.7575
1060.7479
1374.1911
854.6349
%
m/z

G$_5$ GCMS

G_6 Spectra

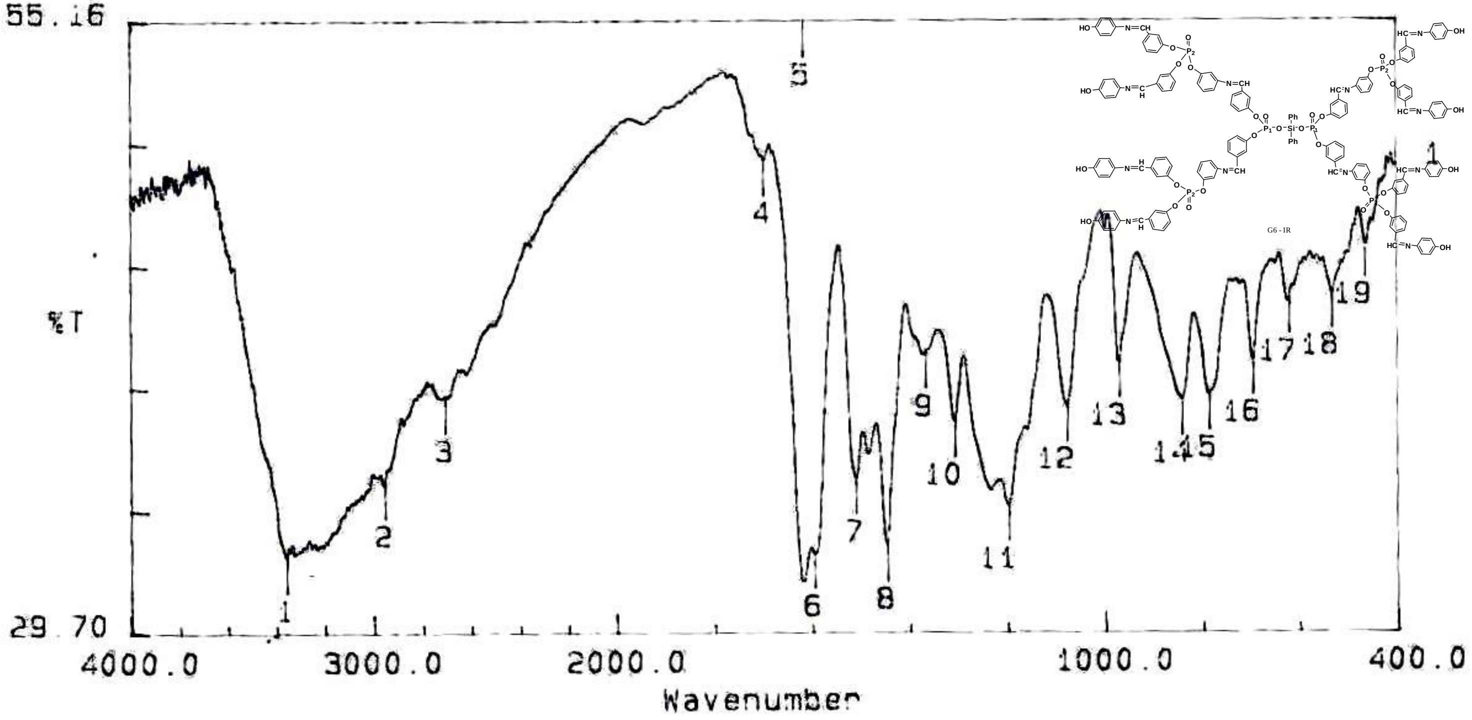

Condition

upper 55.16 lower 29.69 depth 2.00

Peak table

1.	3360.30 (33.0)	2.	2953.28 (36.0)	3.	2706.37 (39.6)	4.	1699.44 (49.4)
5.	1616.49 (31.8)	6.	1597.20 (33.1)	7.	1510.40 (36.2)	8.	1446.74 (33.3)
9.	1367.65 (41.4)	10.	1307.85 (38.5)	11.	1197.90 (35.0)	12.	1078.31 (39.2)
13.	970.28 (41.0)	14.	842.97 (39.4)	15.	787.03 (39.6)	16.	696.37 (41.1)
17.	621.13 (43.6)	18.	534.33 (43.9)	19.	466.82 (46.0)		

G_6 IR Spectrum

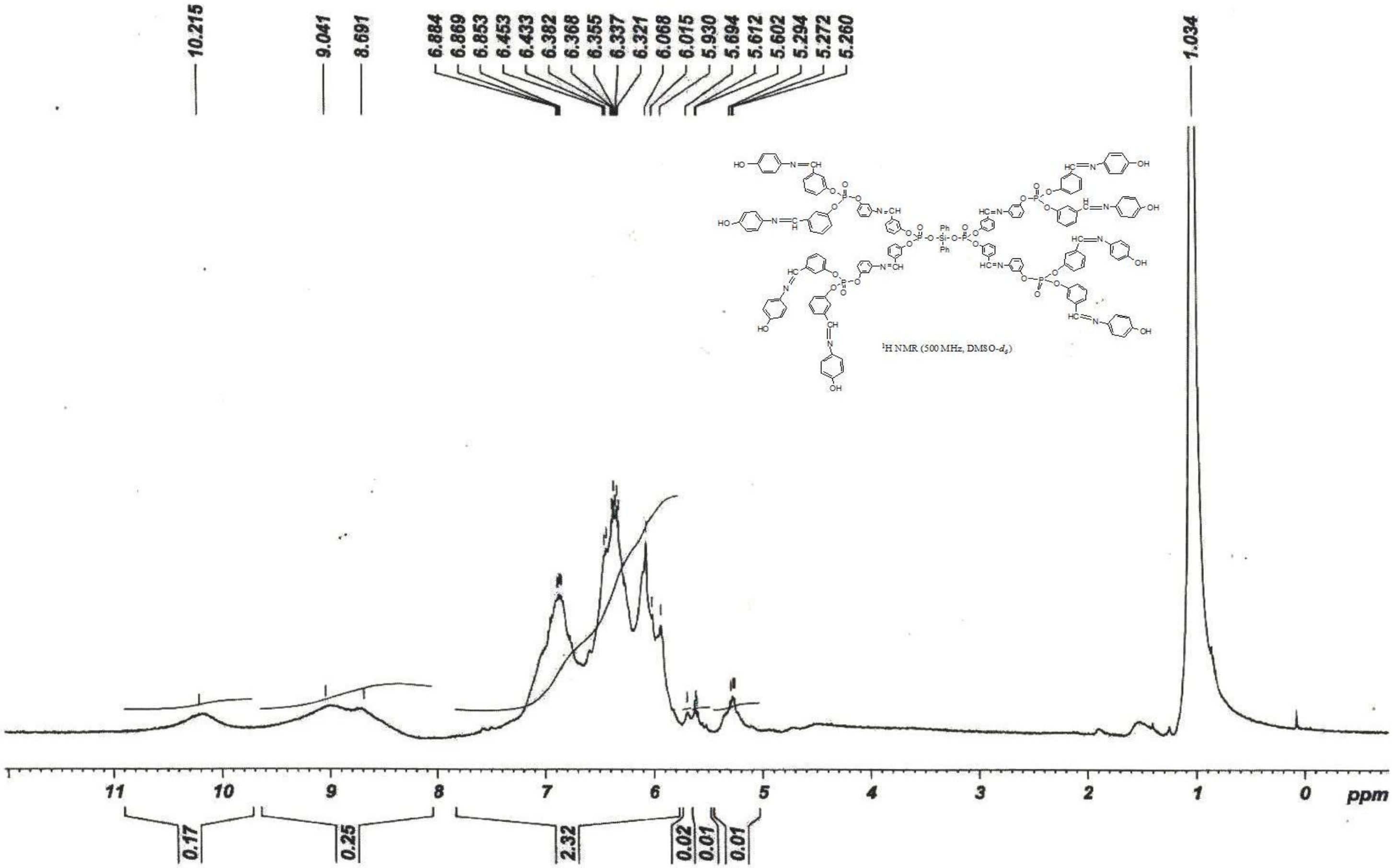

G$_6$ ^{1}H-NMR Spectrum

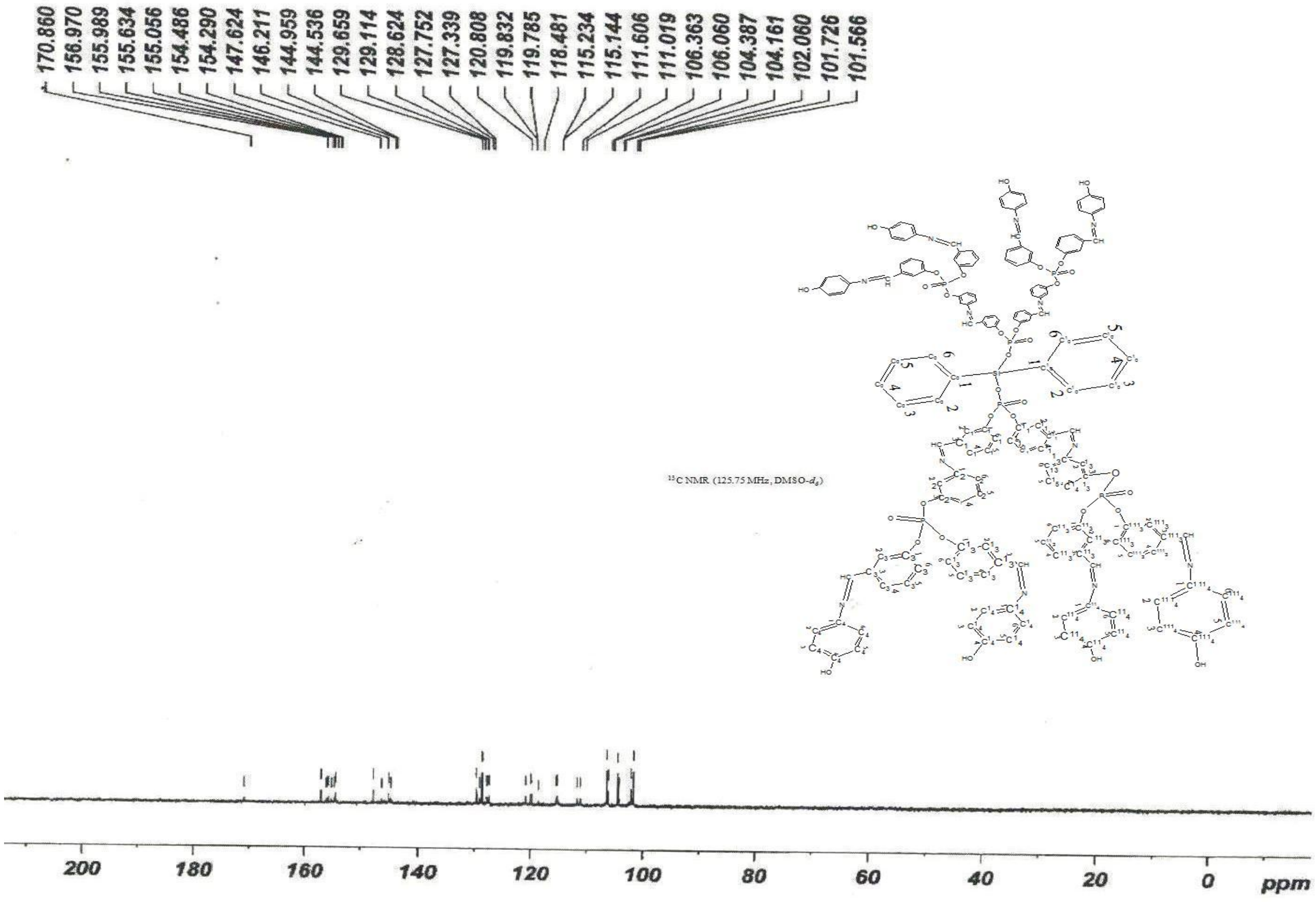

G$_6$ ^{13}C-NMR Spectrum

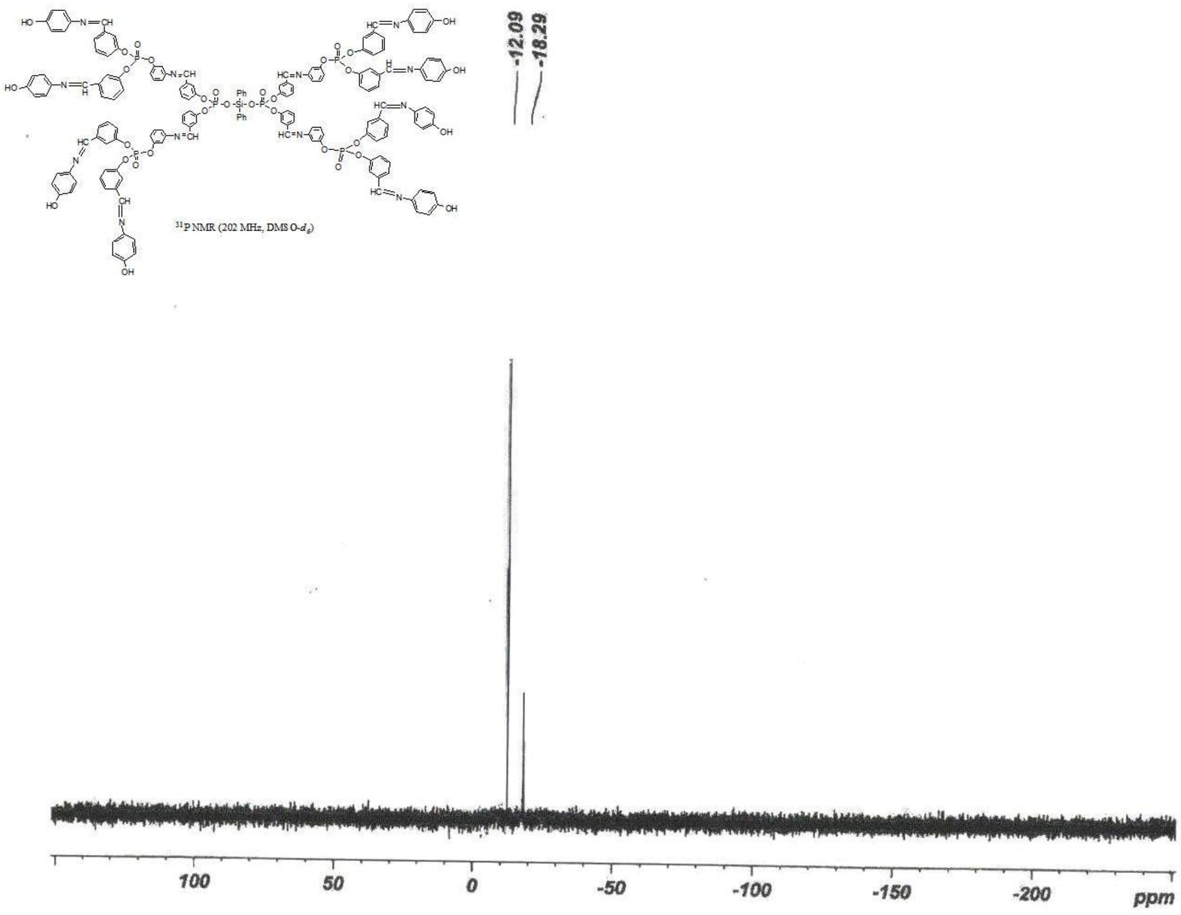

G_6 ^{31}P-NMR Spectrum

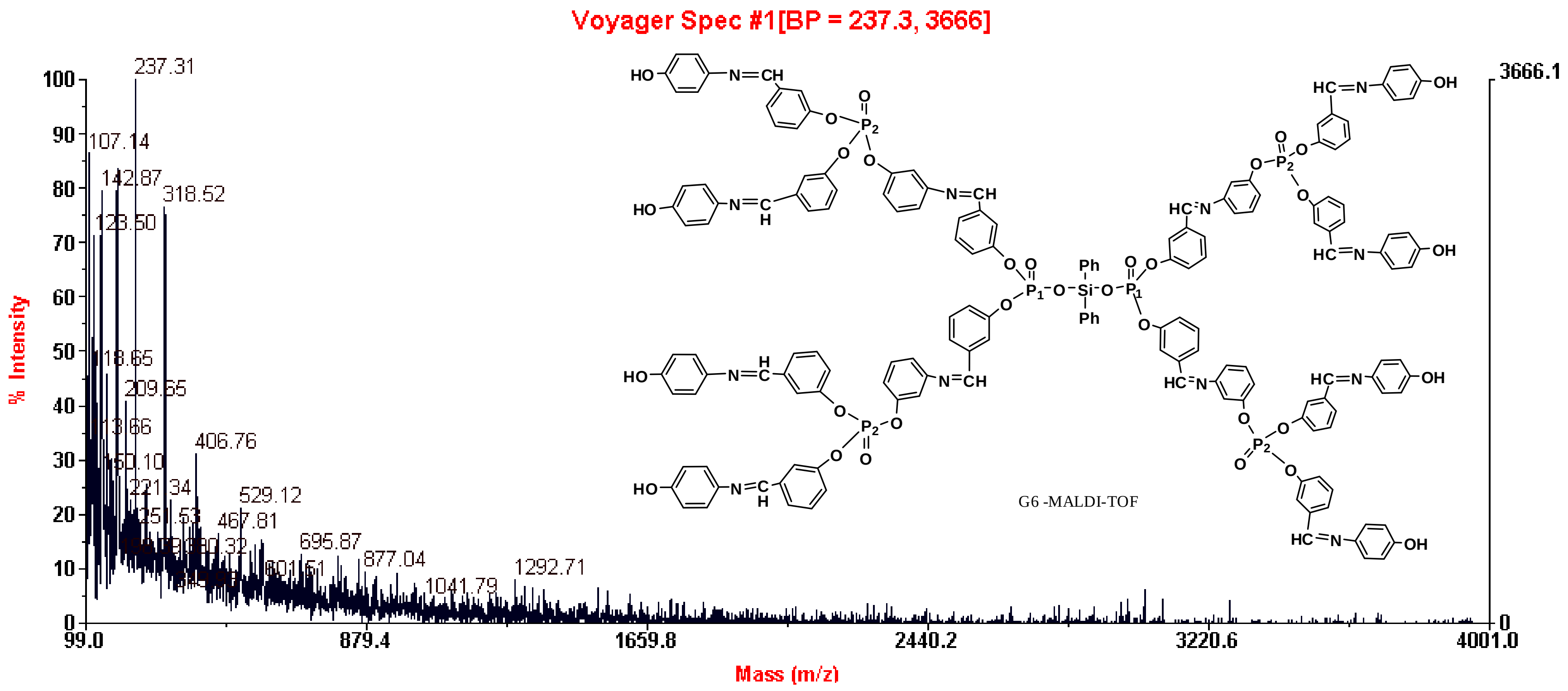
Voyager Spec #1[BP = 237.3, 3666]
% Intensity
Mass (m/z)
237.31
107.14
142.87
318.52
123.50
209.65
406.76
529.12
467.81
695.87
877.04
1041.79
1292.71
3666.1
99.0
879.4
1659.8
2440.2
3220.6
4001.0
G6 -MALDI-TOF

G_6 MALDI-TOF

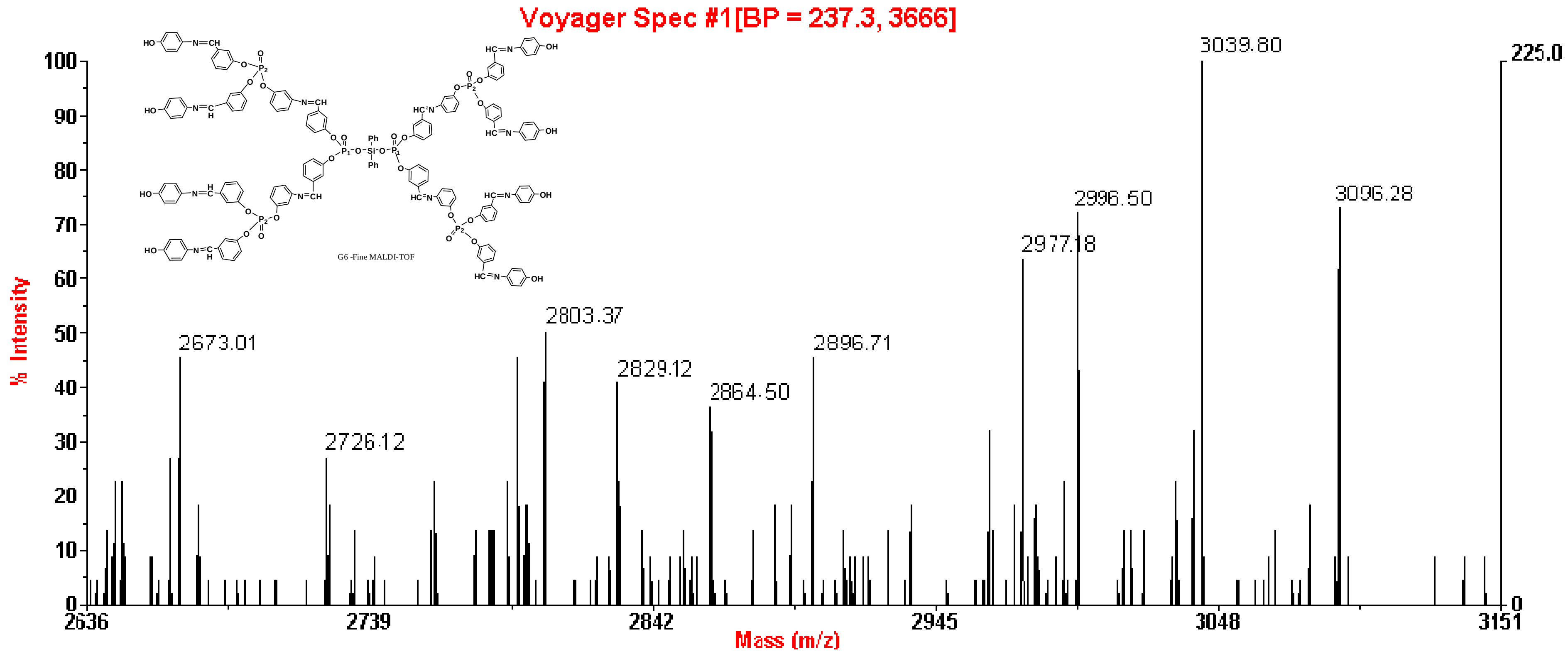

G_6 Fine MALDI-TOF

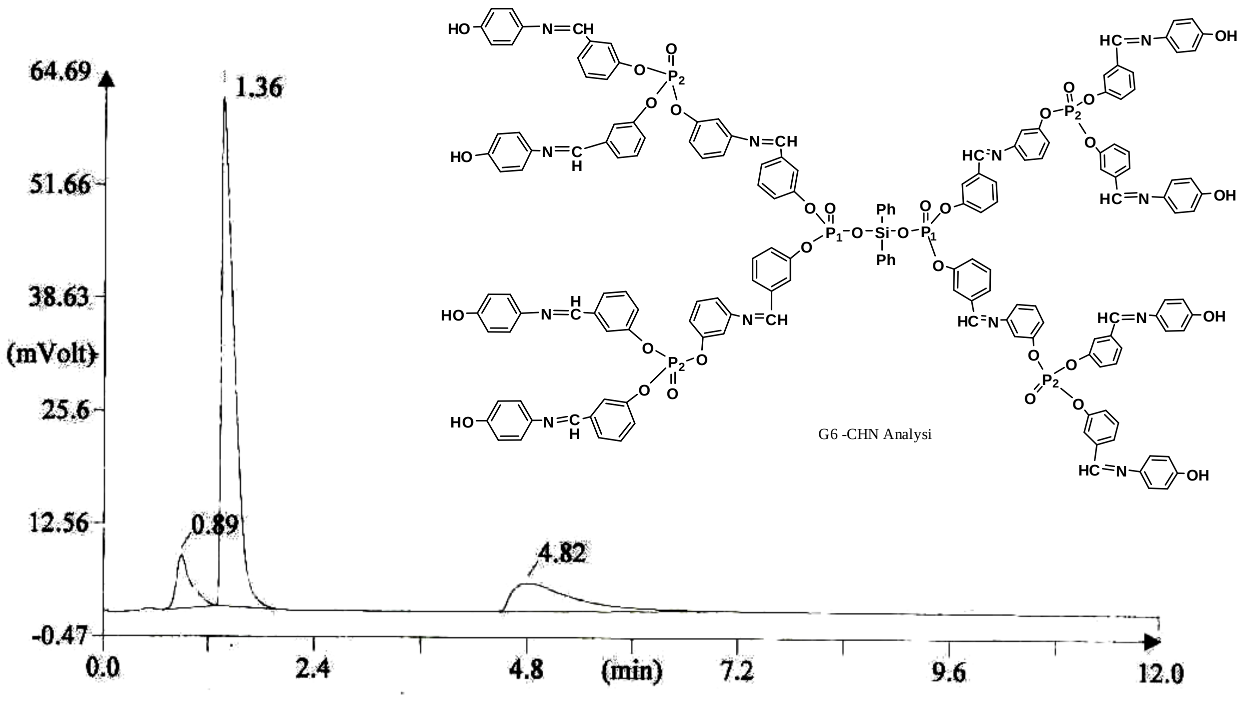

Element Name	Element %	Ret. Time
Nitrogen	5. 61	0. 89
Carbon	66. 25	1. 36
Hydrogen	4. 22	4. 82

G_6 CHN Analysis
